# One‐Step Solid‐State Synthesis of Sandwich‐like, Porous C–SnS_2_ Matrix Composites as Anode Materials for Rechargeable Lithium Ion Batteries

**DOI:** 10.1002/smsc.202500192

**Published:** 2025-07-07

**Authors:** Akzhan Bekzhanov, Irshad Mohammad, Lukas Sallfeldner, Freddy Kleitz, Damian Cupid

**Affiliations:** ^1^ Center for Low‐Emission Transport Vienna AIT Austrian Institute of Technology GmbH 1210 Vienna Austria; ^2^ Vienna Doctoral School in Chemistry (DoSChem) University of Vienna Wahringer Str. 42 1090 Vienna Austria; ^3^ Department of Functional Materials and Catalysis, Faculty of Chemistry University of Vienna Wahringer Str. 42 1090 Vienna Austria

**Keywords:** batteries, composites, polyacrylonitriles, porous, solid‐states, syntheses, tin sulfides

## Abstract

SnS_2_ (tin disulfide) is a promising anode active material for lithium‐ion batteries (LIBs) due to its high theoretical capacity and low material cost. Conventional synthesis methods, such as solvothermal, hydrothermal, and solid‐state, require long synthesis times, the use of solvents and surfactants, and several separation steps. However, the preparation of coated SnS_2_ composites using liquid media is even more complex, requiring suitable precursors, compatible solvents, and potentially several steps. In the present work, a one‐step solid‐state method is developed to synthesize SnS_2_ particles sandwiched in a porous polyacrylonitrile (PAN)‐based matrix phase (C–SnS_2_) for use as anode active materials for LIBs. The as‐synthesized materials exhibit a reversible capacity of 720 mAh g^−1^ after 100 cycles when tested versus Li/Li^+^. The performance of this SnS_2_‐based anode active material is compared to that prepared by the solid‐state heat treatment of SnS_2_, both with and without PAN. The structure, morphology, chemistry, and electrochemical properties of these compounds are established and comprehensively compared to each other. The observed superior cycling stability and rate capability of the sandwich‐like C–SnS_2_ are attributed to its phase purity and its incorporation in a porous, conductive, carbonized PAN matrix.

## Graphical abstract

A one‐step solid‐state method is developed to synthesize SnS_2_ particles, which are embedded in sandwich‐like, porous polyacrylonitrile (PAN)‐based matrix phase (C–SnS_2_) for use as anode active materials for rechargeable lithium‐ion batteries. The performance of this SnS_2_‐based anode active material is compared to that of materials prepared by solid‐state heat treatment of hydrothermally synthesized SnS_2_, both with and without PAN.

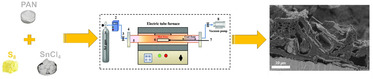

## 1 Introduction

In the search for storage solutions that enable the use of energy from renewable sources, lithium‐ion batteries (LIBs) have emerged as a cornerstone technology for electric vehicles and stationary storage system applications. Currently, graphite‐based carbonaceous materials are still used as anode active materials for LIBs owing to their good electrochemical properties. However, they are limited by the low theoretical Li‐ion storage capacity of 372 mAh g^−1^.^[^ ^1^, ^2^, ^3^, ^4^ ^]^ The ongoing demand for LIBs with higher energy densities drives the pursuit of novel electrode materials exhibiting higher capacities, low degradation rates, and stability with state‐of‐the‐art electrolytes. Among these materials, tin disulfide (SnS_2_), which crystallizes in the CdI_2_‐type structure, has gained interest because of its high theoretical capacity, the abundance of Sn and S, and its low environmental impact.^[^ ^5^, ^6^, ^7^, ^8^, ^9^ ^]^ However, intrinsic challenges such as poor electrical conductivity, volume expansion, low first‐cycle Coulombic efficiency, and severe pulverization during cycling have hindered its practical implementation and upscaling.^[^ ^10^ ^]^

To address these obstacles, extensive research works have focused on the development of composite electrode materials.^[^ ^5^, ^11^, ^12^, ^13^, ^14^, ^15^, ^16^, ^17^, ^18^, ^19^, ^20^, ^21^, ^22^ ^]^ Among them, the integration of carbonaceous species has proven particularly promising, as they can facilitate electronic transport. For SnS_2_, carbon coatings would offer a synergistic approach to harnessing the intrinsic electrochemical properties of SnS_2_ while simultaneously addressing its inherent drawbacks.^[^ ^23^, ^24^, ^25^, ^26^ ^]^ However, the integration of a porous matrix to embed the active material particles can further lead to improved energy storage performance by enhancing electronic and ionic transports, buffering volume changes, and reinforcing the structural stability of the composite material.^[^ ^4^, ^27^, ^28^ ^]^ Nevertheless, one of the main challenges of C–SnS_2_ remains establishing a simple and efficient synthesis pathway that can be industrially upscaled.

Many works in the literature report on the wet‐chemical synthesis of pure and carbonaceous SnS_2_ using solvothermal and hydrothermal methods.^[^ ^5^, ^7^, ^8^, ^12^, ^15^, ^16^, ^29^ ^]^ These techniques first start with the dispersion and/or dissolution of the Sn‐, S‐, and C‐ precursors in a liquid medium, followed by mixing and a thermal treatment. However, only a limited number of studies discussed the solid‐state synthesis of SnS_2_ and C–SnS_2_, not requiring the first dissolution/dispersion step. One work on the solid‐state synthesis of SnS_2_/C nanospheres used tin, sulfur, and carbon precursors, sealed in a quartz ampule under vacuum, and then subjected to heat treatment. This procedure was performed to enable the samples to attain thermodynamic equilibrium with the self‐developing gas phase during the heat treatment.^[^ ^30^ ^]^ The as‐resulting SnS_2_/C nanospheres were investigated as anodes for sodium‐ion batteries, and no experiments were performed for LIB applications.

Some research works also reported solid‐state methods, however, solely for the synthesis of SnS_2_.^[^ ^31^, ^32^, ^33^ ^]^ Xiao et al. presented a synthesis method based on the phase reactions of Sn, S, and NH_4_Cl^[^ ^31^ ^]^ and mentioned the key role of ammonium chloride in favoring the formation of SnS_2_ in the liquid–solid reaction, which occurs upon heat treatment. In a later work, Xiao et al. used a molten salt reaction between SnCl_2_·2H_2_O and thiourea in air to synthesize SnS_2_.^[^ ^33^ ^]^ However, Xiao et al. did not test the electrochemical performance of the SnS_2_ compounds.

This contribution describes a one‐step procedure for the synthesis of C–SnS_2_ sandwich‐like composites starting from a solid‐state mixture of tin (IV) chloride, elemental sulfur (S_8_), and polyacrylonitrile (PAN) as precursors. As control experiments, conventional SnS_2_ was also synthesized via the hydrothermal method^[^ ^29^ ^]^ and subjected to the same final heat treatment temperature as for the C–SnS_2_ samples (H–SnS_2_). In addition, further experiments were performed using mechanical mixtures of SnS_2_ and PAN, which were also heat‐treated at the same final temperature as the C–SnS_2_ sample. Since phase purity, chemical composition, particle morphology, surface chemistry, and interface properties are factors that should be taken into account to understand the behavior of electroactive materials, the structure–property–performance relationships of the C–SnS_2_, H–SnS_2,_ and C–Sn_*x*_S_*y*_ materials were established using bulk, near‐surface, and surface characterization techniques combined with electrochemical characterization. Raman spectroscopy, X‐ray photoelectron spectroscopy (XPS), and powder X‐ray diffraction (PXRD) were used to deliver quantitative and qualitative analyses of the synthesized powders, the initially charged/discharged electrodes, and postmortem surface analysis of the cycled electrodes. Additionally, the interface resistance and charge transfer kinetics of the materials were investigated by electrochemical impedance spectroscopy (EIS) during the first discharge cycle. In general, the superior cycling stability and rate capability of the C–SnS_2_ sample compared to the others investigated in this work can be attributed to the conductivity of the porous, PAN‐based matrix, which embeds the SnS_2_ particles in a sandwich‐like structure to buffer the volume changes during cycling.

## 2 Results

### 2.1 Physicochemical Characterization

**Scheme 1:**
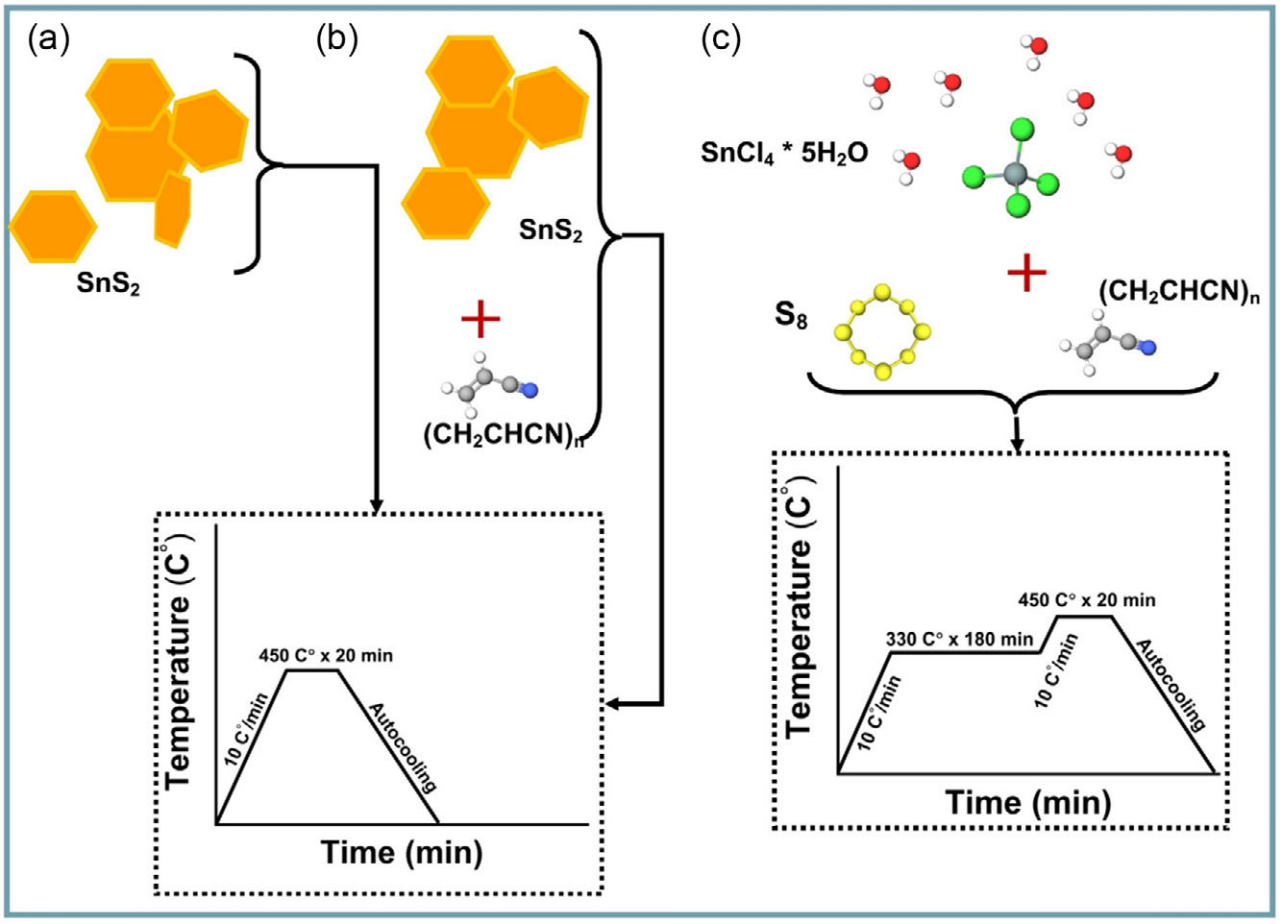
Synthesis route procedure of a) H–SnS_2_, b) C–Sn_*x*_S_*y*_, and c) C–SnS_2_.

**Figure 1:**
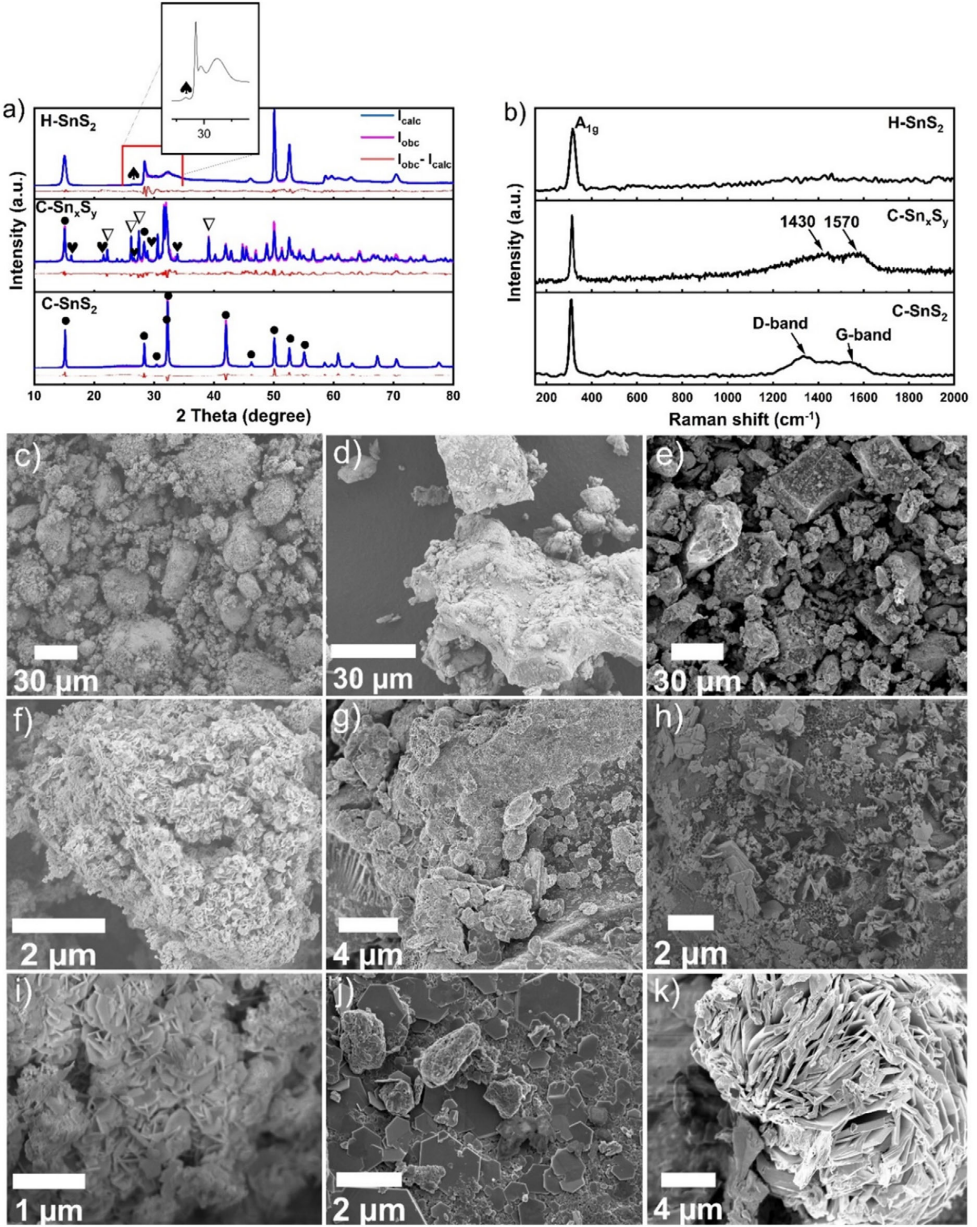
a) Powder XRD patterns of synthesized pristine powders H‐SnS_2_, C‐Sn_*x*_S_*y*_, and C‐SnS_2_. b) Raman spectroscopy analysis of the as‐synthesized H‐SnS_2_, C‐Sn_*x*_S_*y*_, and C‐SnS_2_ pristine powders. SEM images are shown for c,f,i) the H‐SnS_2_, d,g,j) C‐Sn_*x*_S_*y*_, and e,h,k) C‐SnS_2_ synthesized powders.

**Figure 2:**
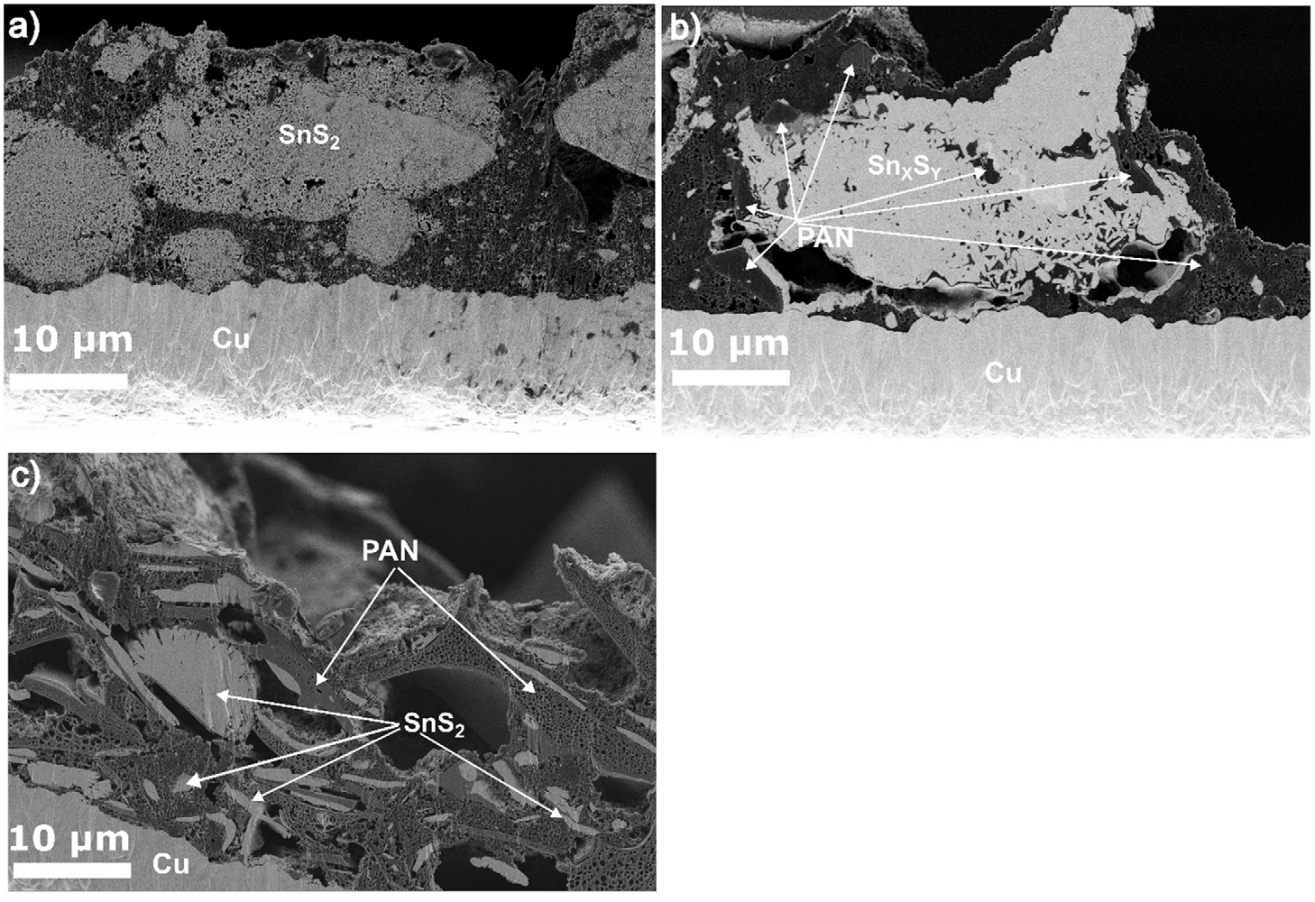
Cross‐sectional microscopy of the pristine electrodes a) H‐SnS_2_, b) C‐Sn_*x*_S_*y*_, and c) C‐SnS_2_.

The synthesis of C–SnS_2_, C–Sn_*x*_S_*y*_, and H–SnS_2_ is schematically illustrated in **Scheme** **1**. After thermal treatment in argon atmosphere, the H–SnS_2_ sample contained SnS_2_ (*P‐3 m 1* space group) as the main phase with a 90% phase fraction and 10% of the metastable cubic SnS zincblende phase (see **Figure** **1**a and **Table** **1**).^[^ ^34^, ^35^ ^]^ The [111] crystal plane of SnS was detected at 26.6° (2 theta), while the reflections associated with the other crystal planes overlapped with the dominant reflections of the [100] and [101] crystal planes of SnS_2_ (see Figure 1a onset). In addition, the small size of the SnS crystals and their small fraction led to comparatively reduced diffraction intensities compared to SnS_2_. The XRD analysis of the C–Sn_*x*_S_*y*_ sample revealed a significant change in the phase composition as a result of heating SnS_2_ with PAN. The structural refinement of C–Sn_*x*_S_*y*_ shows the presence of SnS_2_ (*P‐3 m 1*), Sn_2_S_3_ (*P n m a*), and SnS (*P n m a*), as shown in **Figure** **2**a (reference diffractograms are in Figure S1, Supporting Information).^[^ ^36^, ^37^ ^]^ On the other hand, the Rietveld analysis of the measured PXRD pattern of the C–SnS_2_ sample indicated the presence of only SnS_2_ (*P‐3 m 1)*. The calculated unit cell parameters of the as‐determined phases, their crystallite sizes, phase amounts, atomic concentration (%), and *R*_wp_ values from the Rietveld refinements are compiled in Table 1.

**Table 1:** Refinement values of the powder XRD patterns of Figure 1a.

Sample Name:	Phase fraction, [%]	SnS_2_, space group, unitcell parameters, [Å]	SnS, space group, unitcell parameters, [Å]	Sn_2_S_3_, space group, unitcell parameters, [Å]	R_wp_ [%]	Crystal sizes, [Å]	Sn/S atom. conc. [%]
H‐SnS_2_	SnS_2_ (≥90)	*P*‐3 m 1 a=b=3.622(1), c=5.927(2)	(metastable cubic zincblende)	–	15	182	66.4/33.6
	SnS (≤10)					20–40	
C‐Sn_*x*_S_*y*_	SnS_2_ (39.2)	P‐3 m 1 a=b=3.6476(3), c=5.909(1)	P n m a (62) a=11.217(1) b=3.9998(4) c=4.3023(5)	P n m a (62) a=8.871(3) b=3.7500(8) c=14.024(4)	6.5	334	71.64/28.4
	SnS (41.5)					723	
	Sn_2_S_3_ (19)					618	
C‐SnS_2_	SnS_2_ (100)	P‐3 m 1 a=b=3.6491(1), c=5.9032(2)	**–**	**–**	7.6	556	65/35

Detailed studies on the thermal degradation of PAN report that in the temperature range of 150–350 °C, inter‐ and intramolecular cyclization and laddering of PAN occur, where gaseous species such as hydrogen cyanide (HCN) and ammonia (NH_3_) evolve as byproducts owing to a partial aromatization and conjugation of bonds.^[^ ^38^, ^39^, ^40^ ^]^ Presumably, in our isothermal step at 330 °C, due to gases formed during the decomposition of PAN, hydrogen sulfide and other sulfidic compounds can form at the surface of the SnS_2_ particles, leading to their reduction and the resulting phase transformations of the C–Sn_*x*_S_*y*_ composite. However, during the synthesis of C–SnS_2_, the evolved gases appear to be advantageous for the formation of phase pure SnS_2_. For example, experiments were performed where the masses of SnCl_4_.5H_2_O and sulfur were kept constant at 3 and 1.5 g, respectively, but the amount of PAN was changed (0, 0.1, 0.2, and 0.3 g). When 0 g of PAN was used, no solid phases were formed. However, as the amount of PAN increased, higher amounts of product were formed (see Figure S3, Supporting Information). This indicates that the NH_3_ and HCN gases, which form from the decomposition of PAN, may interact with the SnCl_4_ to form intermediate products such as ammonium chloride and hydrogen chloride, which enable the formation of SnS_2_ during the solid–liquid phase reaction with sulfur at 330 °C.

Raman spectroscopy analysis of the as‐synthesized H–SnS_2_, C–Sn_*x*_S_*y*_, and C–SnS_2_ pristine powder samples is shown in Figure 1b. The Raman spectra of the three samples show a significant peak at 314 cm^−1^, which is attributed to the A_1g_ vibration mode of SnS_2_. In theC–SnS_2_ sample, two broad peaks at 1340 cm^−1^ and 1540 cm^−1^ are observed. The peak at 1540 cm^−1^ is associated with the in‐plane bending motion or stretching of doubly bonded carbon atoms (G‐band) within the graphene layers of graphite. The peak at 1340 cm^−1^ is related to the ring breathing mode, typically associated with disorder in the stacked graphene layers. For the C‐Sn_*x*_S_*y*_ sample, the D‐ and G‐band peaks were observed at 1430 and 1570 cm^−1^, respectively.^[^ ^41^, ^42^ ^]^ The shift in the D‐band and G‐band positions in the Raman spectra of C–Sn_*x*_S_*y*_ and C–SnS_2_ may be due to the different heating profiles used. In our experiments, the C–SnS_2_ sample was subjected to longer total heat treatment times with an intermediate step at 330 °C, which could result in a more complete decomposition of the PAN precursor. Therefore, in the C–Sn_*x*_S_*y*_ sample, the shift in the D‐band to 1430 cm^−1^ is most likely due to the presence of *trans*‐(CH)_n_ moieties at grain boundaries, which may originate from the less complete thermal decomposition of PAN, whereas the G‐band at 1570 cm^−1^ can be associated with the stretching motion of sp^2^‐hybridized C‐atoms.^[^ ^43^ ^]^

Figure 1 also shows the scanning electron microscopy (SEM) images of the H‐SnS_2_, C–Sn_*x*_S_*y*_, and C–SnS_2_ as‐synthesized powders. As shown in Figure 1i, agglomerates of platelets are visible at high magnification for H–SnS_2_. The size of the single hexagonal platelets was estimated to be around 200 nm, whereas that of the agglomerates are in the range of 5–40 μm (see Figure 1c,f). According to Figure 1d,g, C–Sn_*x*_S_*y*_ is primarily composed of nonuniform agglomerates, some of which appear to be covered by a layer. Fine hexagonal platelets are also observed on the surface of secondary particles (see Figure 1j). Similarly, the C–SnS_2_ sample demonstrated a broad distribution of secondary particles with sizes up to 40 μm (see Figure 1e). These agglomerates are composed of platelets in the size range of 1–7 μm, which are embedded in a matrix phase (see Figure 1h,k).

Further insights into the SnS_2_‐based products and electrode morphologies are provided by the cross‐sectional images shown in Figure 2. In all samples, tin‐sulfide‐based particles can be observed on the microscopic scale (Figure 2a,b,c). However, in the samples that were heat‐treated with PAN, a porous matrix is additionally present. In C–Sn_*x*_S_*y*_, the larger secondary particles are nonhomogeneously covered by the porous, PAN‐based matrix (Figure 2b). In the C–SnS_2_ sample, however, the matrix phase embeds the smaller SnS_2_ particles to yield a sandwich‐like structure that also contains micro‐sized voids. In both cases, the resulting microstructures appear to be strongly influenced by the nonuniform wetting of the particle surfaces by the PAN polymer during the thermal treatment.

Thermogravimetric analysis (TGA) was performed on SnS_2_, PAN, and on 85–15 wt% mixture of SnS_2_ and PAN to determine the weight loss of the samples during heat treatment in inert atmosphere. These results are shown in Figure S2, Supporting Information, and the measured weight losses up to 450 °C are listed in Table S1, Supporting Information. The PAN, which was used in this work, exhibited an onset temperature for mass loss at ≈290 °C. According to data from the literature, this is related to the cyclization of the nitrile groups, and further weight loss at higher temperatures is associated with the removal of NH_3_, HCN, and N_2_ with a 51.6% mass loss at the end of the isothermal step at 450 °C.^[^ ^38^, ^39^, ^40^, ^44^ ^]^ The pure SnS_2_ sample exhibited a total mass loss of 6.1% up to 450 °C, which is usually associated with the loss of sulfur and the concomitant reduction of Sn^4+^ to Sn^2+^. The mass loss of a mixture of PAN and SnS_2_ containing 15 wt% PAN and 85 wt% SnS_2_ was measured to be 9.1%. However, the theoretical mass loss of this mixture, calculated based on the measurements of the individual components, should be 12.8%. Therefore, the lower observed mass loss may be attributed to the suppression of the sulfur evolution, which can enhance the tendency for the formation of phase pure SnS_2_ during thermal treatment at 450 °C.

**Figure 3:**
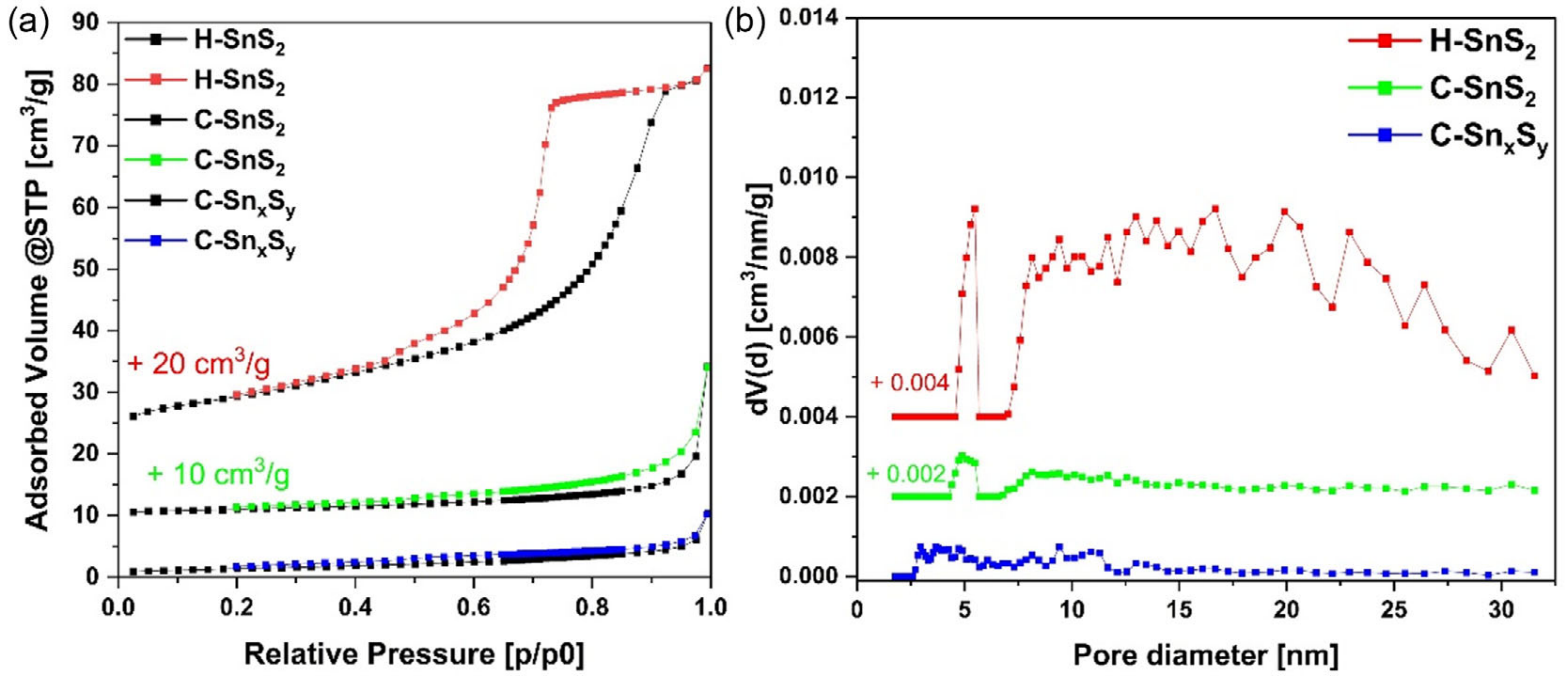
a) N_2_ physisorption isotherms measured at 196 °C and b) respective NLDFT pore size distributions (PSDs), for the as‐synthesized pristine powder samples.

Nitrogen adsorption–desorption isotherm analysis was performed to characterize the surface area, pore structure, and porosity of the materials, which could affect the electrochemical performance of the materials. The N_2_ physisorption isotherm measurements indicated that the specific surface areas (SSAs) of H–SnS_2_, C–Sn_*x*_S_*y*_, and C–SnS_2_ were 35.5, 5.2, and 4.4 m^2^ g^−1^, respectively, and the total pore volume values remained below 0.1 cm^3^ g^−1^ for all samples (see **Figure** **3**a,b and **Table** **2**). The samples C–Sn_*x*_S_*y*_ and C–SnS_2_ exhibited similar N_2_ sorption isotherms with no hysteresis loop, leading to an average pore diameter of 2.9 nm and ≈4.9 nm, respectively. The H‐SnS_2_ exhibited a pronounced capillary condensation step with a wide H2‐type^[^ ^45^ ^]^ hysteresis loop, and the calculated average pore diameter was approximately ≈16 nm.

**Table 2:** SSA and TPV based on the N_2_ physisorption analysis.

Sample	SSA [m^2^ g^−1^]	TPV [cm^3^ g^−1^]
H‐SnS_2_	35.5	0.097
C‐Sn_*x*_S_*y*_	5.2	0.016
C‐SnS_2_	4.4	0.037

**Figure 4:**
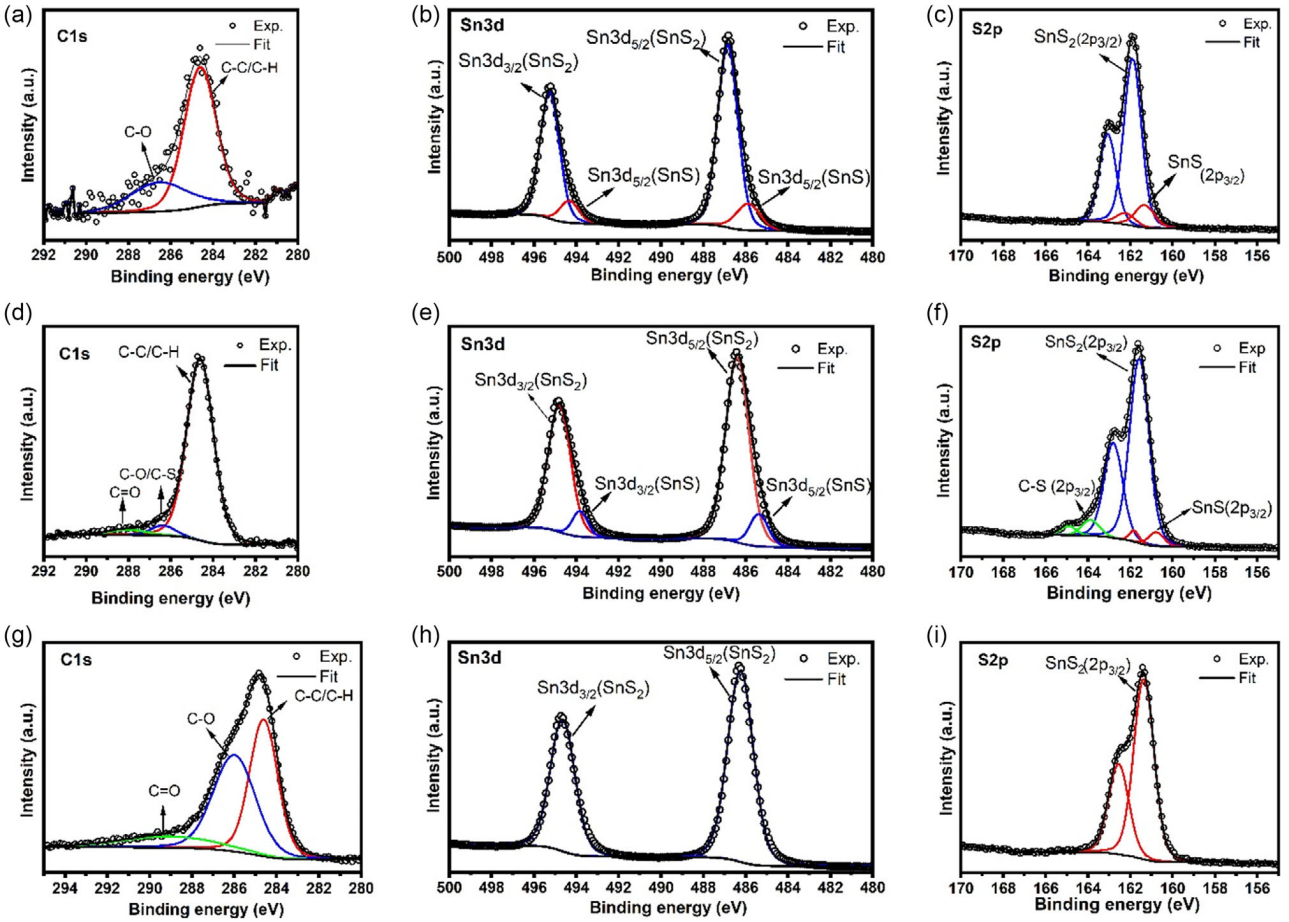
a) C1s, b) Sn 3 d, and c) Sn 2p XPS spectra of H‐SnS_2_. d) C1s, e) Sn 3 d, and f) Sn 2p XPS spectra of C‐Sn_*x*_S_*y*_. g) C 1s, h) Sn 3 d, and i) S 2p XPS spectra of C‐SnS_2_ with tolerance (σ ≈ 0.3 eV).

The surface chemical compositions of the H‐SnS_2_, C‐Sn_*x*_S_*y*_, and C‐SnS_2_ samples were assessed by XPS analysis (**Figure** **4**). Figure 4a shows the high‐resolution C 1s spectrum of the H‐SnS_2_ sample. This spectrum contains two, low‐intensity peaks at 284.6 and 286.7 eV, which belong to the C‐C/C‐H and C‐O groups, respectively. Due to their low intensities relative to the background, these peaks may be associated with impurities and contaminants from air that are localized at the surfaces of the sample. The Sn 3 d spectrum (Figure 4b) for the same sample contains doublets that are characteristic for Sn 3d_3/2_ and Sn 3d_5/2_ electrons. Via peak deconvolution, two sets of doublets could be identified. The binding energies of the doublets at 486.8 and 495.2 eV with higher intensities are associated with Sn^4+^ in SnS_2_,^[^ ^29^ ^]^ whereas those at the lower binding energies of 485.8 and 494.2 eV and with lower intensities are attributed to Sn^2+^ from SnS.^[^ ^46^ ^]^ The core‐level S 2p spectrum shows two peaks that are associated with the S 2p_3/2_ and S 2p_1/2_ states, respectively. Using peak deconvolution, the binding energies of the S 2p peaks at 161.8 and 163.0 eV are associated with S^2−^ in SnS_2_,^[^ ^29^ ^]^ whereas the lower‐intensity doublets at 161.3 and 162.2 eV originate from S^2−^ in SnS^[^ ^46^ ^]^ (see Figure 4c). The C 1s core spectrum (Figure 4d) of the C‐Sn_*x*_S_*y*_ sample shows three peaks at 284.6, 286.4, and 288.0 eV, which are attributed to typical C–C/C–H, C‐O/C‐S, and C=O bonds, respectively.^[^ ^47^ ^]^ The intensities associated with the C 1s spectrum from C‐Sn_*x*_S_*y*_ are higher than those for the H‐SnS_2_ sample, indicating that they are not only associated with impurities and contaminants originating from contact with air but also have strong contributions resulting from the thermal decomposition reaction of PAN. According to the peak‐fitting of the Sn3d spectrum (Figure 4e), one set of doublets is detected at slightly higher binding energies (486.4 and 494.8 eV), representing a higher oxidation state of Sn^4+^, corresponding to SnS_2_.^[^ ^48^, ^49^, ^50^ ^]^ However, a second set of doublets with low intensities for Sn 3d_5/2_ (485.5 eV) and Sn 3d_3/2_ (494.5 eV) with an energy gap of 8.5 eV could also be observed, suggesting the presence of Sn^2+^.^[^ ^49^, ^50^ ^]^ In the S 2p spectrum (Figure 4f), two broad peaks of S 2p_3/2_ at 161.5 eV and S 2p_1/2_ at 162.6 eV were clearly observed, belonging to S^2−^ species of SnS_2_.^[^ ^48^ ^]^ Additionally, two doublets at low‐ and high‐binding‐energy ranges corresponding to SnS (160.8 and 161.8 eV)^[^ ^46^ ^]^ and C‐S (163.9 and 164.9 eV) were detected, respectively.^[^ ^51^, ^52^ ^]^ However, no peaks could be used to identify only Sn_2_S_3_, since both the Sn^2+^ and Sn^4+^ oxidation states are present in this mixed‐valency compound,^[^ ^53^ ^]^ which causes an overlap with the characteristic peaks for SnS_2_ and SnS, respectively. As shown in Figure 4g for the C‐SnS_2_ sample, the C 1s spectrum of C‐SnS_2_ shows three characteristic peaks at 284.6, 286, and 289 eV, which correspond to the C—C/C—H, C—O, and C—C/C=O group bonds, respectively. Figure 4h shows that the Sn 3d spectrum exhibits a typical pair of Sn 3d_3/2_ and Sn 3d_5/2_ peaks at 494.7 and 486.5 eV, respectively. No additional doublets could be found via peak deconvolution. In addition, the S 2p spectrum, shown in Figure 4i, contains peaks at 162 and 163.2 eV,^[^ ^29^ ^]^ which can be assigned to the binding energies of S 2p_3/2_ and S 2p_1/2_, respectively. The peak positions are identical with the values for S^2−^ in SnS_2_.

According to the XPS evaluations, the C–S bond is observed in the C 1s and S 2p spectra of the C‐Sn_*x*_S_*y*_ sample. However, XPS could not identify the presence of this bond in the C‐SnS_2_ sample. This could be attributed to the different sources of sulfur used in the precursor materials. In the case of C‐Sn_*x*_S_*y*_, sulfur atoms are chemically bound to Sn in the SnS_2_ lattice and reacts directly with the PAN at higher temperatures to produce a sulfur‐containing matrix phase. On the other hand, due to the high volatility of elemental sulfur in the absence of strong chemical affinity or confinement, it does not react directly with carbon in the PAN‐based matrix, and no C‐S bonds are observed in the C‐SnS_2_ sample.

**Scheme 2:**
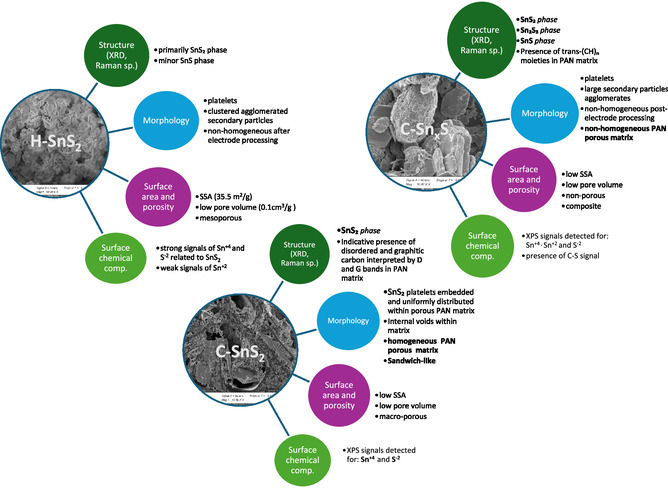
Summary of physicochemical characterization results represented as a radial diagram for H‐SnS_2_, C‐Sn_*x*_S_*y*,_ and C‐SnS_2_ samples.

**Scheme** **2** summarizes the physicochemical characterization results for the H‐SnS_2_, C‐Sn_*x*_S_*y*,_ and C‐SnS_2_ samples. The C‐SnS_2_ sample exhibits a sandwich‐like structure with SnS_2_ particles and large voids embedded in a porous PAN‐based matrix. In C‐Sn_*x*_S_*y*_, the PAN‐based matrix is porous, but it is nonhomogeneously distributed around the larger secondary particles. The variations in the D‐band and G‐band positions in the Raman spectra of C‐Sn_*x*_S_*y*_ and C‐SnS_2_ may be due to the heat treatment temperature and duration applied. In the case of C‐Sn_*x*_S_*y*_, a greater presence of *trans*‐(CH)_n_ moieties indicates a lower degree of decomposition of PAN. The C‐SnS_2_ sample was subjected to longer total heat treatment times, allowing a more complete decomposition. Additionally, the XRD and XPS measurements confirmed the phase composition and their elemental oxidation states, respectively. Additionally, the Sn 3d and S 2p spectrum analyses are in good agreement with the phases detected by Rietveld refinement analysis of the H‐SnS_2_, C‐Sn_*x*_S_*y*_, and C‐SnS_2_ samples.

### 2.2 Electrochemical Characterization

**Figure 5:**
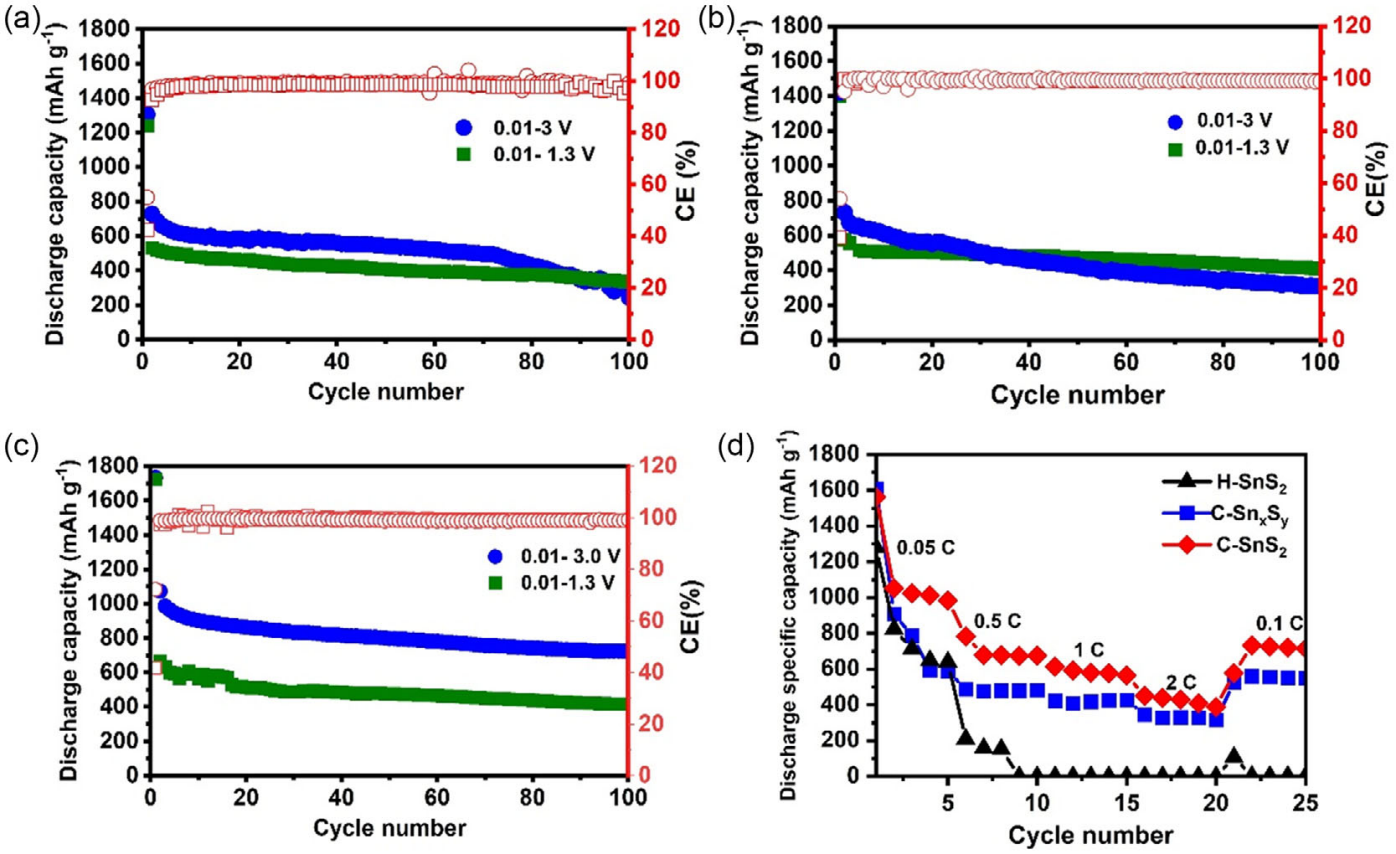
Galvanostatic cyclic performances in potential limitations 0.01–3 V and 0.01–1.3 V for a) H‐SnS_2_, b) C‐Sn_*x*_S_*y*,_ and c) C‐SnS_2_, and the rate capability performance of the three samples in a 0.01–3.0‐V potential range in d).

The cycling performances of the three samples were tested at a current density of 0.1 A g^−1^ in the 0.01–1.3 V and 0.01–3.0 V potential windows (see **Figure** **5**a,b,c). The initial Coulombic efficiencies (CEs) and the delivered capacities for the samples can be found in **Table** **3**. The best cycling performance was achieved at 720 mAh g^−1^ for the C‐SnS_2_ sample after 100 cycles in the potential window between 0.01 and 3 V versus Li/Li, as shown in Figure 5c. The C‐Sn_*x*_S_*y*_ and H‐SnS_2_ samples demonstrated poor cycling performances in the higher potential window up to 3 V versus Li/Li, with reversible capacities of 310 and 243 mAh g^−1^ after 100 cycles, respectively. However, the cycling performance in the lower potential window (0.01–1.3 V) was found to be similar for the C‐Sn_*x*_S_*y*_ and C‐SnS_2_ samples with a retained capacity of 411 and 416 mAh g^−1^, respectively. For the H‐SnS_2_, the capacity after 100 cycles in the potential window 0.01–1.3 V versus Li/Li^+^ was found to be 76 mAh g^−1^ lower than for the other samples that were cycled in the same potential range.

**Table 3:** Initial CE of the first cycle and capacity retention after 100 cycles for the H–SnS_2_, C–Sn_*x*_S_*y*_, and C–SnS_2_ samples in 0.01–1.3 V and 0.01–3 V potential windows.

Sample	Initial CE [%],		Capacity retention after 100 cycles [mAh g^−1^]	
	0.01–1.3 V	0.01–3 V	0.01–1.3 V	0.01–3 V
H‐SnS_2_	42	55	335	243
C‐Sn_*x*_S_*y*_	39	54	411	310
C‐SnS_2_	41	68	416	720

The rate capabilities of the three samples are shown (see Figure 5d), where current densities of 0.05, 0.5, 1, 2, and 0.1 A g^−1^ were sequentially applied. The C‐SnS_2_ sample delivered capacities of 983, 677, 565, 390, and 715 mAh g^−1^ at the end of each rate step, respectively. A similar trend was observed for the C‐Sn_*x*_S_*y*_ although the discharge capacities are on average 25% lower than that of the C‐SnS_2_ samples. On the other hand, poor rate capability performance was observed for the H‐SnS_2_ sample, with full capacity loss at 1 C without any recovery when cycling was performed at 0.1 C after the high‐rate testing.

**Figure 6:**
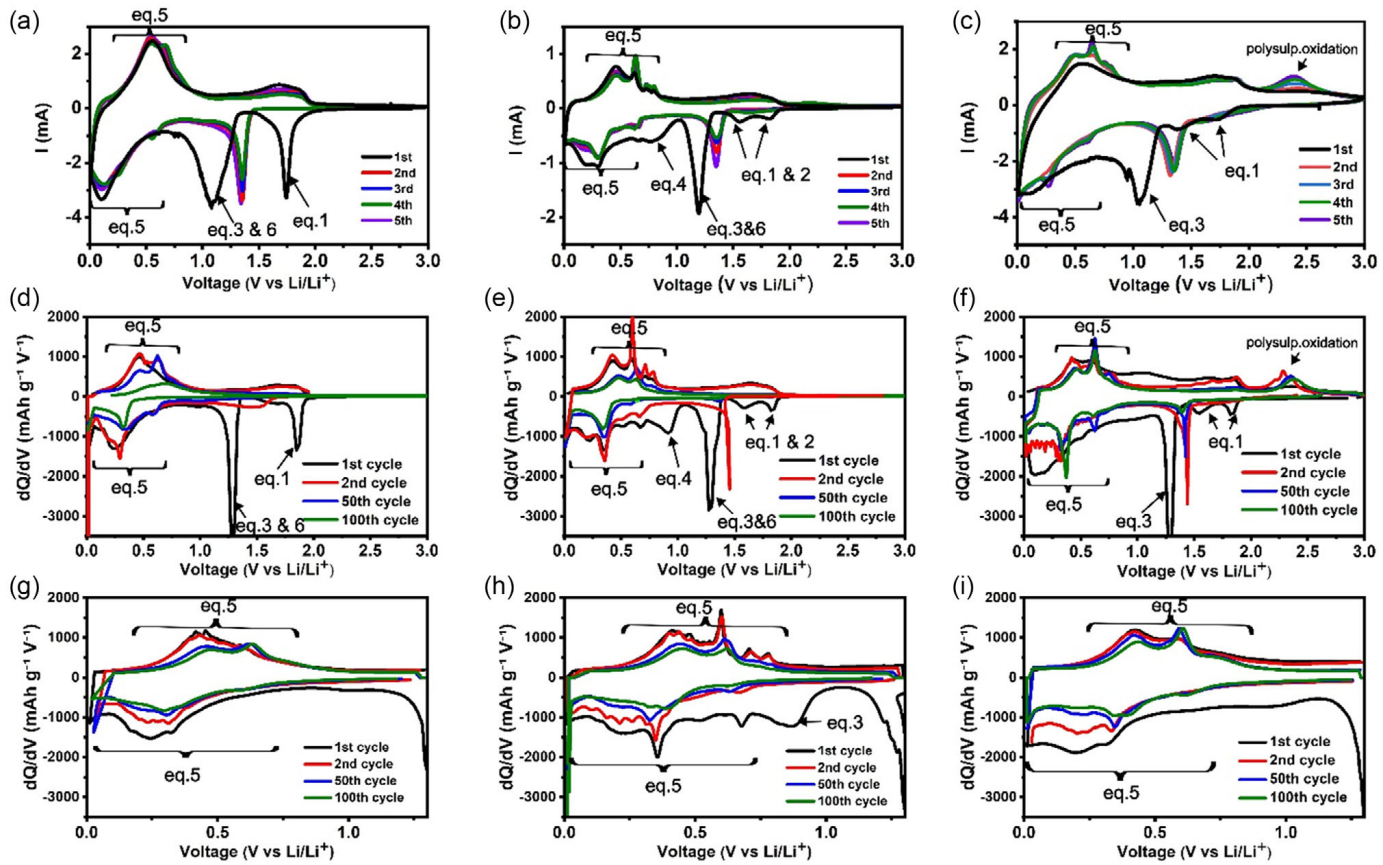
CV of H‐SnS_2_, C‐Sn_*x*_S_*y*,_ and C‐SnS_2_ samples at a scan rate of 0.2 mV s^−1^ in a), b), and c), respectively. The DC plots at the selected 1st, 2nd, 50th, and 100th cycles in 0.01–3‐V and 0.01–1.3‐V potential windows for d,g) H‐SnS_2_, e,h) C‐Sn_*x*_S_*y*_, and f,i) C‐SnS_2_, respectively.

In this study, both cyclic voltammetry (CV) and differential capacity (DC) curves were performed to identify the characteristic redox peaks (see **Figure** **6**a–i). CV is susceptible to distortions caused by factors such as electrolyte resistance, charge transfer kinetics (when rate‐limiting), and solid‐state diffusion limitations. In contrast, DC analysis is more robust against such distortions. Although DC measurements also reflect the impact of diffusion and kinetic limitations, since the voltage must diverge from equilibrium to sustain a constant current, they provide a clearer isolation of electrochemical phenomena such as phase transitions, overpotentials, and charge/discharge asymmetry, without artifacts introduced by ohmic drops.

In the H‐SnS_2_ sample, one large reduction peak with an onset at 1.95 V is observed for the intercalation reaction (Equation 1). However, for C‐Sn_*x*_S_*y*_ and C‐SnS_2_, two peaks are measured in this region. The first peak has an onset at 1.9 V for both C‐Sn_*x*_S_*y*_ and C‐SnS_2_, whereas the second peak has an onset at 1.67 V for C‐Sn_*x*_S_*y*_ and an onset of 1.55 V for C‐SnS_2_. For both C‐Sn_*x*_S_*y*_ and C‐SnS_2_, the first two peaks are associated with a two‐step intercalation process of Li^+^ into the SnS_2_ lattice, where in the first step, Li^+^ occupies the octahedral sites between the SnS_2_ layers, and in the second step, Li^+^ starts to occupy the tetrahedral positions within the layers. This two‐step intercalation process is clearly observed in the CV for the samples containing the PAN‐based matrix, suggesting that the matrix provides additional electronic and ionic conductivities to improve the reaction kinetics, thereby allowing a better resolution of the peaks associated with the intercalation reaction. Conversely, for the H/SnS_2_ sample, where no PAN‐based matrix is present, only one large peak is observed for the intercalation process. These observations are also confirmed by the DC data, where one large peak is measured for the intercalation reaction of Li^+^ into SnS_2_ (see Figure 6d) and two peaks are observed for the intercalation mechanism for the C‐Sn_*x*_S_*y*_ and C‐SnS_2_ samples with the PAN‐based matrix (see Figure 6e,f, respectively). Similar phenomena were also reported by,^[^ ^54^ ^]^ who showed that for an SnS_2_ sample, only one peak at 1.7 V was observed for the interaction reaction, whereas for the SnS_2_/rGO sample with higher conductivity, two peaks at 1.82 and 1.6 V, respectively were measured for the two‐step intercalation process.

Sn_2_S_3_ was also found in the PXRD measurements of the C‐Sn_*x*_S_*y*_ sample, and due to the presence of Sn^4+^ ions and vacancies for Li^+^ in their crystal lattices, Sn_2_S_3_ may also be expected to intercalate Li^+^ ions with an accompanying reduction of Sn^4+^ to Sn^2+^ (equation 2). A detailed study of the Sn_2_S_3_ lithiation mechanism reported via density functional theory (DFT) by Chakraborty et al.^[^ ^55^ ^]^ states that the intercalation of Li in Sn_2_S_3_ occurs at 1.55 V and the Li hosting limit in Li_*x*_Sn_2_S_3_ is x ≈ 1. In the C‐Sn_*x*_S_*y*_ sample, both effects (lithiation of the tetrahedral sites of SnS_2_ at lower potentials and lithiation of Sn_2_S_3_) may overlap each other. According to Chakraborty et al., one may expect the possible nondestructive intercalation of lithium into the crystal lattice of Sn_2_S_3_ due to the presence of large interstitial sites in the crystal structure of Sn_2_S_3_ and the small ionic radius of lithium (equation 2).

In the CV scans, the broad reduction peaks at 1.1 V for H‐SnS_2_ and C‐SnS_2_ have onsets at 1.3 and 1.24 V, respectively, whereas the peak at 1.2 V for C‐Sn_*x*_S_*y*_ has an onset at 1.3 V. These peaks correspond to the formation of Sn and Li_2_S via the conversion reaction according to Equation 3 to Equation 6. These conversion reaction peaks are more distinctly observed at 1.3 V versus Li/Li^+^ for all samples in the DC curves plotted against potential during the first discharge, as shown in Figure 6d,e,f. Interestingly, an experimental study by Chen et al. on the sodiation of Sn_2_S_3_ reports the presence of reaction peaks at 1.0 and 0.8 V during the conversion of Sn_2_S_3_.^[^ ^56^ ^]^

In all CV scans, a reduction peak at 0.6 V and a broader peak at 0.3 V, along with their paired oxidation peaks at 0.45 V and 0.65 V, are observed, which correspond to the alloying and dealloying stages of Li‐Sn, respectively. These lithiation and delithiation stages are also evident in the DC curves for the three samples (see Figure 6d–i). The C‐Sn_*x*_S_*y*_ sample exhibits an additional reduction peak near 0.9 V during the first cycle in both the CV and DC curves (see Figure 6b,e,h). This feature may potentially be attributed to the phase conversion of Li_*x*_Sn_2_S_3_ into Li_2_S and Sn. A broad anodic peak is observed at 1.9 V in the first CV scan of all samples and the first and second charge cycles of the DC curves, indicating the partial conversion of Sn and Li_2_S back to SnS_2_. Additionally, an anodic peak at 2.3 V, distinguishable only for the C‐SnS_2_ sample, may correspond to the transformation of Li_2_S into polysulfides or sulfur (see Figure 6c,i).^[^ ^57^, ^58^ ^]^ The redox peaks associated with mentioned specific reaction mechanisms for all samples can be seen as plateaus in initial discharge/charge versus potential curves in Figure 5d, as well

(1) $${\text{SnS}}_{2}+{\text{xLi}}^{+}+{\text{xe}}^{–}\to {\text{Li}}_{x}{\text{SnS}}_{2}$$

(2) $${\text{SnS}}_{1.5}+{\text{xLi}}^{+}+{\text{xe}}^{–}\to {\text{Li}}_{x}{\text{SnS}}_{1.5}\;({\text{for Sn}}_{2}S_{3})$$

(3) $${\text{Li}}_{x}{\text{SnS}}_{2}+(\text{4 – x}){\text{ Li}}^{+}+(\text{4 – x}){\text{ e}}^{–}\to {\text{Sn}+2\text{Li}}_{2}S$$

(4) $${\text{Li}}_{x}{\text{SnS}}_{1.5}+{\text{3Li}}^{+}+{\text{3e}}^{–}\to {\text{Sn}+1.5\text{Li}}_{2}\text{S }({\text{for Sn}}_{2}S_{3})$$

(5) $${\text{Sn}+\text{xLi}}^{+}+{\text{xe}}^{-}⇌{\text{Li}}_{x}\text{Sn}$$

(6) $${\text{SnS}+2\text{Li}}^{+}+{\text{2e}}^{-}\to \;{\text{Sn}+\text{Li}}_{2}S$$

**Figure 7:**
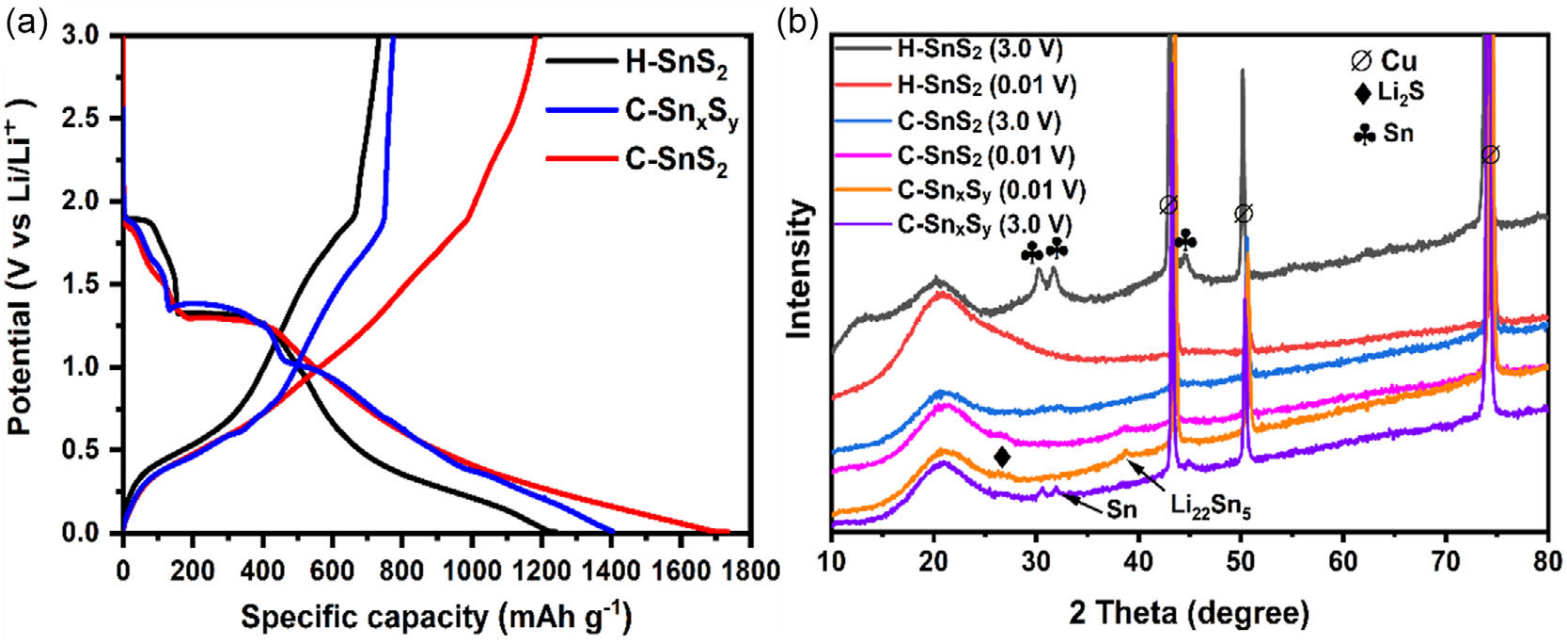
The discharge/charge specific capacity versus potential graph of the 1st cycle is given in (a). b) Postmortem ex situ powder XRD patterns of H‐SnS_2_, C‐Sn_*x*_S_*y*_, and C‐SnS_2_ electrodes at charged and discharged states during the 1st cycle.

To confirm the conversion and alloying reactions, according to the listed Equation (3), (4), (5), (6), postmortem ex situ XRD studies were performed after the 1^st^ cycle (see **Figure** **7**b and the reference patterns in Figure S1, Supporting Information). The common feature of a tetragonal Sn phase was found for the three samples charged up to 3.0 V, although its corresponding XRD peaks at 31° and 32° (2 theta) were broadened and had low intensity for the C‐SnS_2_ sample. Interestingly, a lithiated Li_22_Sn_5_ phase was found at a potential of 0.01 V after discharge of the C‐SnS_2_ and C‐Sn_*x*_S_*y*_ samples.^[^ ^59^ ^]^ The [111] plane of the Li_2_S phase was detected at 27° (2 theta) only for the composite samples (C‐Sn_*x*_S_*y*_, C‐SnS_2_), as shown in Figure 7b. However, due to the small crystallite size of the Li_2_S and the high background, the peaks associated with the [111] plane at 27° (2 theta) could not be clearly observed for the H‐SnS_2_ sample.

**Figure 8:**
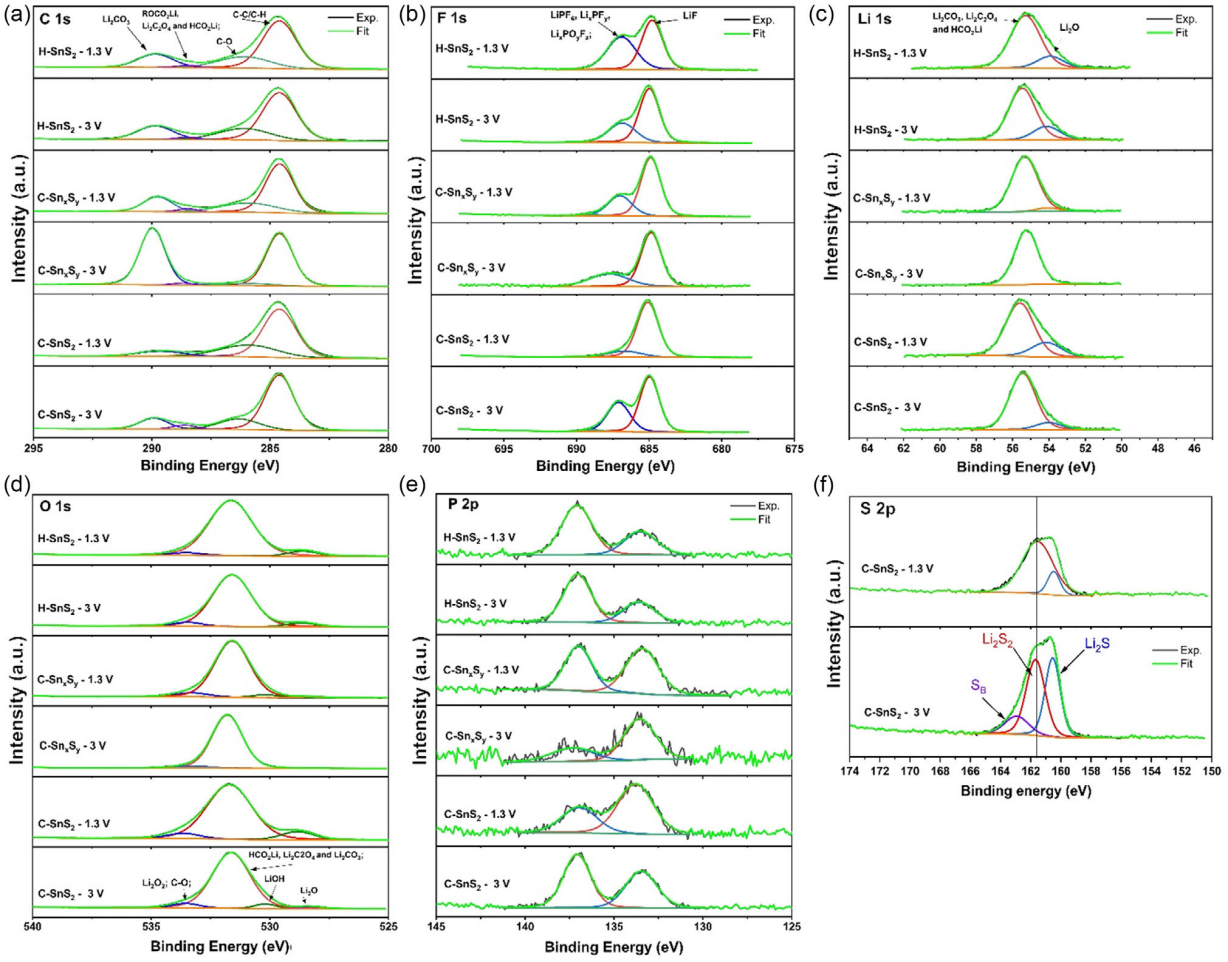
Postmortem XPS spectra for the C a) 1s, b) F 1s, c) Li 1s, d) P 2p, and e) O 1s elemental regions of the H‐SnS_2_, C‐n_*x*_S_*y*_, and C‐SnS_2_ electrodes after 100 cycles. The spectral data for each sample were collected within the potential ranges of 0.01–1.3 V and 0.01–3 V, corresponding to the respective elements cycled. f) Further, continuous depth profile etching for the cycled C‐SnS_2_ electrodes was performed to investigate the S 2p spectral region in both potential window regions.

Postmortem XPS was performed on the electrodes after 100 cycles up to 3.0 and 1.3 V versus Li/Li^+^, respectively, to analyze the surface composition of the solid‐electrolyte interphase (SEI) layer in the charged state after cycling in the different potential windows (see **Figure** **8**). The summarized binding energy (BE) values and elemental atomic concentrations based on the XPS survey and peak fitting ratios are listed in **Table** **4**.

**Table 4:** Postmortem XPS analysis of the C 1s, F 1s, li 1s, O 1s, P 2p, and S 2p spectral assignments for the H–SnS_2_, C–Sn_*x*_S_*y*,_ and C–SnS_2_ samples. The relative peak positions (BE), atomic concentrations (calculated by the areal integration analysis), possible chemical compounds, and bonds of the listed elements are given. The reference BE values for the chemical species were compared and considered from the following literature.^[^ ^62^, ^63^, ^64^, ^65^, ^66^, ^67^ ^]^ The maximum absolute deviation value is shown with tolerance (*σ* ≈ 0.3 eV).

Name	C 1s peaks [eV],concent. [%]	Chemicalcompoundsand/or bonds	F 1s peaks [eV]),concent. [%]	Chemicalcompoundsand/or bonds	Li 1s peaks [eV],concent. [%]	Chemicalcompoundsand/or bonds	O 1s peaks [eV], concent. [%]	Chemicalcompoundsand/or bonds	P 2p peaks [eV], concent. [%]	Chemicalcompoundsand/or bonds
**atomic concent. [%]**	**29.64**		**3.4**		**41.56**		**24.8**		**0.6**	
H‐SnS_2_ (0.01–1.3 V),	284.6 (17.5%) 286.08 (6.36%) 288.5 (0.6%) 289.85 (5.17%)	C‐C/C‐H; C‐O; Li_2_C_2_O_4_ and HCO_2_Li; Li_2_CO_3_;	684.8 (1.8%) 686.9 (1.61%)	LiF; LiPF_6_, Li_*x*_PF_y_, Li_*x*_PO_y_F_z_, (p‐FC_6_H_5)3_ PO;	53.92 (7%) 55.3 (34.5%)	Li_2_O; Lithium carbonates;	528.6 (1.41%) 531.63 (22.6%) 533.31 (0.7%)	Li_2_O; HCO_2_Li, Li_2_C_2_O_4_ and Li_2_CO_3_; Li_2_O_2_; C‐O;	133.52 (0.2%) 137.08 (0.4%)	Li_*x*_PF_y_ LiPF_6_
**atomic concent. [%]**	**28.6**		**5.4**		**41.6**		**23.7**		**0.7**	
H‐SnS_2_ (0.01–3 V)	284.6 (16.4%) 286.1 (6.6%) 288.5 (0.8%) 289.8 (4.8%)	C‐C/C‐H; C‐O; Li_2_C_2_O_4_ and HCO_2_Li; Li_2_CO_3_;	685 (3.6%) 686.85 (1.7%)	LiF; LiPF_6_, Li_*x*_PF_y_, Li_*x*_PO_y_F_z_;	54.13 (8.6%) 55.47 (33%)	Li_2_O; Lithium carbonates;	528.67 (1.3%) 531.6 (21.2%) 533.45 (1.15%)	Li_2_O; HCO_2_Li, Li_2_C_2_O_4_ and Li_2_CO_3_; Li_2_O_2_; C‐O;	133.6 (0.2%) 137 (0.5%)	Li_*x*_PF_y_ LiPF_6_
**atomic concent. [%]**	**32.75**		**4.6**		**35.9**		**26.24**		**0.47**	
C‐Sn_x_S_y_, (0.01–1.3 V),	284.6 (18.95%) 285.9 (7.08%) 288.5 (0.9%) 289.75 (5.8%)	C‐C/C‐H; C‐O; Li_2_C_2_O_4_ and HCO_2_Li; Li_2_CO_3_;	684.9 (3.18%) 687 (1.34%) 689.06 (0.07%)	LiF; LiPF_6_, Li_*x*_PF_y_ and Li_*x*_PO_y_F_z_; Fluoro‐organic (C‐F);	54.07 (1.8%) 55.34 (34.1%)	Li_2_O; Lithium carbonates	528.4 (0.26%) 530.2 (0.91%) 531.6 (23.4%) 533.3 (1.61%)	Li_2_O; LiOH; HCO_2_Li, Li_2_C_2_O_4_, alkyl carbonates and Li_2_CO_3_; C‐O; Li_2_O_2_;	133.4 (0.26%) 137 (0.21%)	Li_*x*_PF_y_ LiPF_6_
**atomic concent. [%]**	**16.85**		**0.56**		**21.35**		**61.6**		**0.05**	
C‐Sn_*x*_S_y_, (0.01–3 V),	284.6 (7.9%) 285.9 (0.6%) 288.7 (0.31%) 289.97 (8%)	C‐C/C‐H; C‐O; ROCO_2_ Li, Li_2_C_2_O_4_ and HCO_2_Li; Li_2_CO_3_;	684.86 (0.38%) 687.8 (0.17)	LiF; Fluoro‐organic moiety (C‐F);	55.23 (21.35%)	Lithium carbonates	531.7 (59%) 533.21 (2.6%)	HCO_2_Li, Li_2_C_2_O_4_ and Li_2_CO_3_; C‐O; Li_2_O_2_;	133.5 137	Li_*x*_PF_y_ LiPF_6_
**atomic concent. [%]**	**34.52**		**10.8**		**40.12**		**14.16**		**0.4**	
C‐SnS_2_, (0.01–1.3 V),	284.6 (21.26%) 285.8 (9.87%) 288.26 (0.7%) 289.7 (2.7%)	C‐C/C‐H; Li_2_CO_3_;	685 (9%) 686.5 (1.8%)	LiF; LiPF_6_, Li_*x*_PF_y_ and Li_*x*_PO_y_F_z_;	54.14 (9%) 55.5 (31%)	Li_2_O; Lithium carbonates	528.82 (1.3%) 531.7 (12%) 533.64 (0.8%)	Li_2_O; HCO_2_Li, Li_2_C_2_O_4_ and Li_2_CO_3_; Li_2_O_2_; C‐O;	133.77(0.26%) 136.94(0.13%)	Li_*x*_PF_y_ LiPF_6_
**atomic concent. [%]**	**36.13**		**4.82**		**35.7**		**22.5**		**0.77**	
C‐SnS_2_, (0.01–3 V),	284.6 (23.8%) 286.3 (6.2%) 288.6 (1.44%) 289.9 (4.6%)	C‐C/C‐H; C‐O; ROCO_2_ Li, Li_2_C_2_O_4_ and HCO_2_Li; Li_2_CO_3_;	685 (2.92%) 687.1 (1.81%) 688.9 (0.07%)	LiF; LiPF_6_, Li_*x*_PF_y_ and Li_*x*_PO_y_F_z_; Fluoro‐organic (CF);	54 (3.9%) 55.4 (31.7%)	Li_2_O; Lithium carbonates (Li_2_CO_3_, Li_2_C_2_O_4_ and HCO_2_Li)	528.34 (0.4%) 530.2 (1%) 531.62 (20%) 533.55 (1.1%)	Li_2_O; LiOH; HCO_2_Li, Li_2_C_2_O_4_ and Li_2_CO_3_ Li_2_O_2_; C‐O;	133.46(0.34%) 137(0.42%)	LiPF _6_

The study focuses on high‐resolution scans of key elemental peaks, such as Li 1s, F 1s, O 1s, P 2p, and C 1s. The decomposition of the LiPF_6_ electrolyte salt and carbonate‐based solvents can be identified mainly by the presence of inorganic LiF and lithium carbonate compounds, respectively. The elemental composition at the surface of the H‐SnS_2_ electrodes that were cycled in both potential windows showed nearly the same species. However, slight increases in LiF and Li_2_O can be observed in the 0.01–3 V cycling window, which can be associated with higher amounts of electrolyte decomposition. The evaluation of the C‐Sn_*x*_S_*y*_ showed distinct differences in oxygenated lithium carbonate species (HCO_2_Li, Li_2_C_2_O_4_, alkyl carbonates, and Li_2_CO_3_), where their concentrations were three times higher in the 0.01–3 V cycling window compared to the 0.01–1.3‐V window. However, the atomic concentrations of F‐containing species decreased from 4.6% in the 0.01–1.3 V cycling window to 0.56% in 0.01–3 V, which indicates lower concentrations of F‐containing species in the SEI formed at the higher voltage cutoff. It can also be observed that the C‐C/C‐H concentrations (which originate largely from carbonaceous species such as carbon black and PAN matrix) decreased 2.4x times from the low to the high potential window range. This indicates that the SEI layer deposits on top of the original, carbon‐containing layer formed from the thermal decomposition of PAN, and a thicker SEI is produced when cycling is performed in the higher potential window. For the C‐SnS_2_ sample, oxygen‐based (Li_2_C_2_O_4_ and HCO_2_Li, Li_2_CO_3_) species increased 1.7x based on the O 1s spectrum, whereas the Li‐containing species LiF and Li_2_O decreased by 3 times from the low to the high potential window, respectively. As also observed for the C‐Sn_*x*_S_*y*_ sample, more decomposition of carbonate solvents and thicker SEI layers are indicated in the higher potential window for C‐SnS_2_. For the composite electrodes containing thermal decomposition productions of PAN, it can be said that the SEI layer contains more LiF and Li_2_O species at lower voltage cutoffs but more lithium carbonate species at the higher potential cutoffs. Fluoroethylene carbonate (FEC) decomposition products were not directly observed in most of the samples, since peaks associated with the fluoro‐organic compound (containing ‐CF_x_ functional groups) usually appear above 290 and 688 eV in the C 1s and F 1s spectra, respectively. The H‐SnS_2_ samples also showed the presence of chemical species similar to the composite samples; however, the ratios of lithium carbonate‐, fluorine‐, and oxygen (Li_2_O)‐based species were found similar in high and low potential cutoffs.

To summarize, the H‐SnS_2_ sample shows no significant changes in the chemical composition of the SEI layer as a result of cycling up to 1.3 and 3.0 V versus Li/Li^+^, respectively. However, both the C‐Sn_*x*_S_*y*_ and C‐SnS_2_ samples exhibit varied concentrations of the chemical species in the SEI layer as a function of the upper cutoff voltage. The concentration of the carbonate‐containing species increases at the higher voltage limit, whereas the signals from the PAN‐based matrix decrease, indicating that the PAN‐based matrix reacts with the electrolyte, and the extent of these reactions is higher at the 3.0‐V voltage cutoff. This strongly indicates that the presence of the PAN‐based matrix surrounding the active material particles influences the chemistry, formation, and growth of the SEI layer.

Additionally, S 2p scans were performed with a 300‐sec depth prob. etching for the C‐SnS_2_ samples charged to 1.3 V and 3 V to elucidate additional oxidation reactions occurring at 2.3 V (see Figure 8f). The S 2p spectrum showed several peaks at 160.5, 161.6, and 163.3 eV at the high potential window cutoff and 160.5 eV and 161.65 eV at the low potential cutoff. These peaks are associated with Li_2_S at 160.5 eV, and 161.6, 163.4 eV with terminal S (S_T_ or Li_2_S_2_) and bridge‐S (S_B_) polysulfide species, respectively. The S_B_ detection at high potential cutoff confirms the incomplete oxidation of lithium sulfide to bridge‐S during the charge step up to 3.0 V versus Li/Li^+^. Huu et al. similarly reported the formation of S_T_, S_B_, and S_8_ species at a 3 V potential cutoff and Li_2_S and S_T_ species at a 0.01 V potential, where nitridic graphene was used as component in the S‐based composite.^[^ ^57^ ^]^

**Figure 9:**
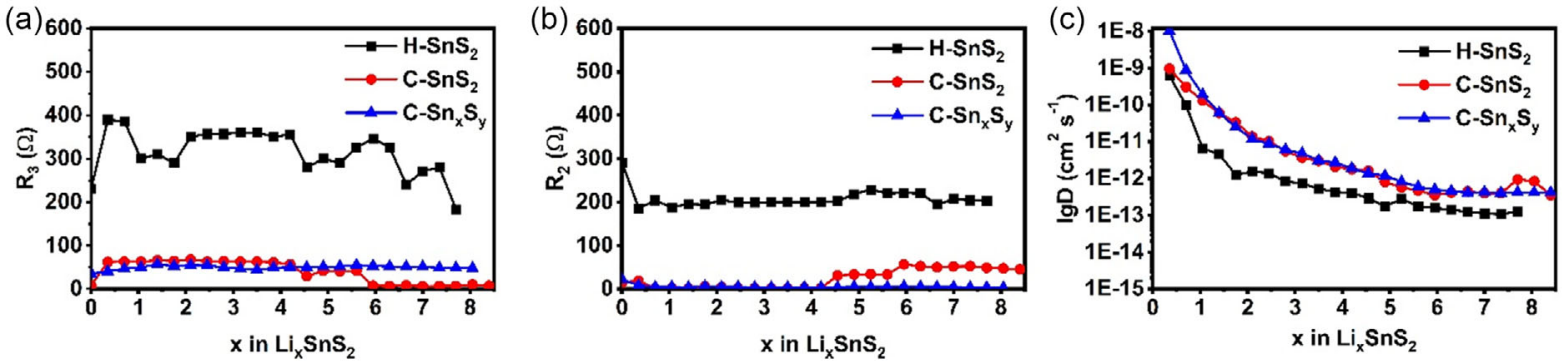
Retrieved charge transfer (R_3_) and interfacial resistance (R_2_) values versus × in Li_*x*_SnS_2_ plotted in a) and b), respectively. (D _Li_^+^) coefficients from in situ EIS spectra were calculated and shown versus *x* in Li_*x*_SnS_2_ in c).

EIS was performed to monitor charge transfer and interfacial resistances during first formation discharge for the H‐SnS_2_, C‐Sn_*x*_S_*y*,_ and C‐SnS_2_ samples. The results are plotted versus the mole of lithiation in **Figure** **9**a,b. The Nyquist plots were fitted using the equivalent circuit shown in Figure S4, Supporting Information, where R_3_ and Q_3_ refer to the charge transfer resistance and capacitance at the electrode/electrolyte interface, R_2_ and Q_2_ refer to the SEI layer resistance and capacitance, respectively, R_1_ is related to the combination of the resistances arising from the current collectors, separator and electrolyte, while the Warburg element (W_4_) is associated with the diffusion of Li^+^ into the bulk material. The charge transfer values are summarized in Figure 9a. The electron charge transfer kinetics are found to be faster for the C‐SnS_2_ and C‐Sn_*x*_S_*y*_ samples (<90 Ohm) and more sluggish for H‐SnS_2_ (≥200 Ohm). While the charge transfer resistances for the H‐SnS_2_ sample vary with the degree of lithiation, they are constant for the C‐Sn_*x*_S_*y*_ and C‐SnS_2_ samples, respectively. The interfacial resistance values were also lower (below <50 Ohm) for C‐Sn_*x*_S_*y*_ and C‐SnS_2_ samples compared to ≈200 Ohm for H‐SnS_2_ samples.

The C‐Sn_*x*_S_*y*_ and C‐SnS_2_ samples exhibit lower charge transfer and SEI resistances compared to the H‐SnS_2_ sample. These can be related to the higher Li^+^ ionic conductivities of the electrolyte/electrode interface, which may be attributed to the higher Li^+^ ionic mobility of the PAN‐based network. Furthermore, the diffusion coefficients of Li^+^ in C‐Sn_*x*_S_*y*_ and C‐SnS_2_ are orders of magnitude higher than those for the H‐SnS_2_ sample, as plotted in Figure 9a, although for all samples, the Li^+^ diffusion coefficients (D _Li_^+^)^[^ ^29^ ^]^ decrease continuously upon lithiation. The higher diffusivities for the C‐Sn_*x*_S_*y*_ and C‐SnS_2_ samples may be a result of the presence of the porous PAN‐based matrix in both samples, which improve the ionic and electronic conductivities of the bulk electrode material.

### 2.3 Discussion

In all samples, higher initial capacities were observed when galvanostatic cycling was performed up to 3.0 V versus Li/Li^+^ compared to 1.3 V. The H‐SnS_2_ sample shows a sudden change in capacity loss in the higher voltage window starting at cycle number ≈70. The XPS investigations confirmed that a thicker SEI was formed on the surface of the H‐SnS_2_ electrodes when cycling was conducted up to the higher voltage cutoff of 3.0 V versus Li/Li^+^ compared to the lower voltage cutoff of 1.3 V versus Li/Li^+^. Since the H‐SnS_2_ sample has an SSA of 35.5 m^2^ g^−1^, which is significantly larger than the SSAs of the other samples, it is highly likely that the electrolyte decomposition is the cause of the strong capacity decay starting at cycle number ≈70.

For the C‐Sn_*x*_S_*y*_ sample, the rate of capacity decay is higher in the larger voltage window over all cycles. The CV curves for H‐SnS_2_ and C‐Sn_*x*_S_*y*_ up to 3.0 V versus Li/Li^+^ show shallow oxidation effects between 1.3 and 2.0 V, indicating that the higher capacities of these samples between 1.3 and 3.0 V versus Li/Li^+^ may be related to oxidation reactions occurring either within the samples or at the electrode/electrolyte interfaces. On the other hand, the C‐SnS_2_ sample exhibits higher capacity in the larger voltage window but the same rate of capacity loss for both potential windows. The CV and DC curves for C‐SnS_2_ show reversible oxidation peaks at ≈2.3 V versus Li/Li^+^. Based on the XPS results, the S 2p depth probing etching confirmed the presence of Li_2_S, S_T_, and S_B_ species at the 3 V potential cutoff but only Li_2_S and S_T_ species at the 1.3 V potential cutoff. However, as no S_8_ signals were observed in the XPS spectra at 3 V versus Li/Li^+^, Li_2_S was not fully oxidized to elemental sulfur. These results indicate that the additional capacity of C‐SnS_2_ between 1.3 and 3.0 V versus Li/Li^+^ is due to the formation of higher‐order polysulfides, and this reaction is reversible.

Phase purity also plays a role in the electrochemical performance of SnS_2_‐based materials. The C‐Sn_*x*_S_*y*_ sample contains large concentrations of SnS and Sn_2_S_3_ in addition to SnS_2_ (39.2 wt%). In a comparative study,^[^ ^60^ ^]^ the authors showed that SnS_2_ outperformed SnS. This was attributed to the higher amount of Li_2_S that is produced during the conversion reaction and the microstructural benefits associated with the intercalation of Li into the SnS_2_ crystal structure prior to conversion. In addition, due to the heterogeneous nature of C‐Sn_*x*_S_*y*_, large mechanical stresses are introduced into the electrode when the three components are lithiated and delithiated. This effect is enhanced at the higher voltage cutoff as revealed in the faster degradation rate.

In addition to phase purity, microstructure has a large influence on the electrode performance. In the microstructure of the C‐SnS_2_ electrode, SnS_2_ particles and platelets are embedded in a porous, PAN‐based matrix in a sandwich‐like structure. This morphology helps to accommodate the volume expansion of the SnS_2_ particles during cycling. This mechanism is further improved by the presence of voids within the PAN‐based matrix, which can also serve to buffer the volume changes of the active material during cycling. For C‐Sn_*x*_S_*y*_, however, the PAN‐based matrix is nonhomogeneously distributed, and the secondary particles are not embedded in the matrix phase. Therefore, this morphology is not as effective at stabilizing the composite structure during cycling. However, the EIS data show that the charge transfer and SEI resistance for the samples that were heat treated with PAN (C‐SnS_2_ and C‐Sn_*x*_S_*y*_) are always lower than those for the PAN‐free, H‐SnS_2_ sample. This indicates that the PAN‐based matrix is both ionically and electronically conductive, which helps to increase the rate capability of C‐SnS_2_ and C‐Sn_*x*_S_*y*_ compared to that of H‐SnS_2_.

## 3 Conclusion

In this work, a one‐step method was developed to synthesize phase pure C‐SnS_2_ composites via a heat treatment of a solid‐state mixture of SnCl_4_·5H_2_O, sulfur (S8), and PAN precursors. The electrochemical performance of C‐SnS_2_ was compared to that of C‐Sn_*x*_S_*y*_ and H‐SnS_2_, which were synthesized with and without PAN, respectively. Our results show that even though both C‐SnS_2_ and C‐Sn_*x*_S_*y*_ contain a PAN‐based matrix, C‐SnS_2_ should be favored because the tin‐sulfide active material is phase pure and evenly distributed within the PAN‐based matrix. This structure is advantageous for accommodating the volume changes of the SnS_2_ active material during cycling. In addition, the intrinsic properties of the PAN‐based matrix improve the ionic and electronic conductivities of the electrode, which results in better rate capability of the materials compared to H‐SnS_2_. Specifically, C‐SnS_2_ exhibited a high reversible capacity of 720 mAh g^−1^ after 100 cycles and retained a reversible capacity of 390 mAh g^−1^ at 2 C‐rate. These conclusions are supported by the cross‐sectional SEM images, which show the embedding of SnS_2_ in the PAN‐based matrix, as well as by the CV and DC curves, which confirm the higher reversibility of the electrochemical redox reactions taking place in C‐SnS_2_.

In general, the electrochemical performance of C‐SnS_2_ is better than that of H‐SnS_2_, which is also better than that of C‐Sn_*x*_S_*y*_. This trend is attributed to the phase fraction of tin disulfide in the active material. This is confirmed by the Rietveld refinement of the powder XRD data, which quantitatively showed that C‐SnS_2_ contained phase pure SnS_2_, H‐SnS_2_ exhibited a SnS_2_‐phase purity of 90 wt%, but C‐Sn_*x*_S_*y*_ exhibited the lowest phase fraction of SnS_2_ (39.2 wt%). Therefore, our results based on XRD and electrochemical cycling data also confirm that attaining phase pure SnS_2_ is essential for ensuring the electrochemical stability and performance of the tin‐sulfide‐based materials.

To conclude, the comparative study of C‐SnS_2_, C‐Sn_*x*_S_*y*,_ and H‐SnS_2_ shows that the successful integration of phase pure SnS_2_ particles into a conductive, porous PAN matrix improves the stability of the tin‐sulfide‐based materials, resulting in significantly improved cycling performance and rate capability. In future, C‐SnS_2_ can also be explored as an alternative anode active material for Na‐ion storage chemistries.

## 4 Experimental Section

4.1

4.1.1

##### Materials

Tin (IV) chloride pentahydrate (SnCl_4_·5H_2_O) from Alfa Aesar (98% purity), thioacetamide (C_2_H_5_NS) from Alfa Aesar (99% purity), sulfur powder (32.07 g mol^−1^) from Sigma Aldrich, PAN (CH_2_CHCN)_n_) fine powder with a mesh size of 50 μm ((molecular weight) 230 kg mol^−1^) from Goodfellow Cambridge Limited, porcelain crucibles, mortar, and pestle, doctor blade coater, carboxymethyl cellulose (CMC) from Alfa Aesar, styrene butadiene rubber (SBR) from JSR Micro, carbon black C65 from Imerys, coin cells (CR2032), Celgard 2325 separators with a 19 mm diameter, Li chip from PI‐KEM, 1 M LiPF_6_ in EC/DMC (ethylene and dimethyl carbonate) with 10 wt% FEC from SOLVIONIC, deionized water (DI), dimethyl carbonate from Sigma Aldrich, ethanol, glass beakers, conductive carbon tape, magnetic stirrers, polycarbonate dome for postmortem XRD, copper foil with 10 μm thickness, 125 mL Teflon‐lined stainless‐steel autoclave, a filter funnel, a vacuum oven, and a Thinky mixer were used.

##### Synthesis Procedure of H‐SnS_*2*_

SnCl_4_·5H_2_O and thioacetamide (C_2_H_5_NS) were dissolved in DI separately in glass beakers. During synthesis, a 1:4 molar ratio of tin chloride to thioacetamide was used corresponding to the best electrochemical cycling performance in our recent work.^[^ ^29^ ^]^ The concentration of the initial tin chloride solution was fixed by dissolving 3 g (8.5 mmol) of tin (IV) chloride in 20 mL of DI and 2.56 g (34 mmol) of thioacetamide in 40 mL of DI water. The mixture of the SnCl_4_ 5H_2_O and C_2_H_5_NS solutions was vigorously stirred for 10 min using a magnetic stirrer, before it was transferred into a 125 mL Teflon‐lined stainless‐steel autoclave. The closed autoclave was heated at 160 °C in an oven for 10 h, after which it was cooled to room temperature. The precipitated products were further separated by centrifugation at 2500 rpm for 5 min (Malvern PANalytical) using a filter funnel. The filtrate was washed 3 times with distilled water to remove any residuals and finally dried in a vacuum at 65 °C overnight. The obtained dry powder of SnS_2_ was transferred to a porcelain crucible and placed into a tubular furnace, which was evacuated and flushed with argon three times. Hereafter, the SnS_2_ sample was annealed for 20 min at 450 °C with a ramp rate of 10 °C min^−1^ under an argon atmosphere (see Scheme 1a).

##### Synthesis Procedure of C‐Sn_*x*_ *S*_*y*_

The dried SnS_2_ powder, which was obtained from hydrothermal synthesis using the procedure above, was mixed with PAN in an 85:15 wt% ratio, respectively, and homogenized using mortar and pestle. Hereafter, the same flushing and heat treatment program were applied for the mixture to obtain the C‐Sn_*x*_S_*y*_ composite (see Scheme 1b). The obtained composite was washed once with an ethanol and twice with DI water to remove any organic residuals and further dried in a vacuum oven at 65 °C through overnight.

##### Synthesis Procedure of C‐SnS_*2*_

Typically, 3 g (8.5 mmol) of SnCl_4_·5H_2_O, 1.5 g (46.8 mmol) of sulfur powder, and 0.2 g of PAN were mixed by mortar and pestle. The mixture was placed in a porcelain crucible and transferred to a tubular furnace. Evacuation and flushing of the tube using argon and the heating ramp rate were applied in the same manner as for previous samples. However, in this procedure, an isothermal step at 330 °C for 180 min was included in the annealing procedure. This was followed by increasing the temperature to 450 °C (10 °C min^−1^ rate) with a holding time of 20 min (see Scheme 1c). The isotherm step at 330 °C was selected to melt the PAN and encourage the formation of SnS_2_, as further explained in the Results. The obtained composite product was washed and dried, similarly to the previous sample. The obtained composite was weighted as 1.15 g.

##### Instrumentation and Characterization

Powder XRD of the samples was measured using X’Pert Pro PANalytical diffractometer with Cu Kα radiation (λ = 1.542 A) over a 2*θ* range of 10–80°. Phase identification and lattice parameter determination were performed via the Rietveld refinement of the measured diffractograms. XPS (Nexsa, Thermofisher) was employed to study the surface chemistry and oxidation states of the powders and electrodes using the following acquisition parameters: Al Kα source gun, spot size 400 μm, pass energy 50 eV, and energy step size 0.1 eV, etching with monoatomic Ar beam at 6000 eV. Prior to XPS analysis, the cycled electrodes were removed from the coin cells and washed with dimethyl carbonate in the glovebox. Afterward, they were placed in a specially built airtight system in the glovebox and inserted directly into the spectrometer to minimize air exposure. The samples were exposed to air around 20 sec max., and the surface of the electrodes was etched 60 s to purify impurities and contaminants from the air. All the obtained XPS spectra were processed with CASA XPS software and calibrated with C 1s signal at a BE of 284.6 eV. Raman spectra were measured using a Horiba Scientific (Jobin‐Yvon‐Horiba) spectrometer with a 532‐nm laser wavelength. SEM (from a Carl Zeiss Supra 40 scanning electron microscope) was used to investigate the morphology of the powders and the electrodes. Samples for surface analysis were prepared by attaching powder or electrodes on the carbon conductive tape. The cross‐sectional electrode samples were prepared using an Ar‐ion mill (Hitachi IM4000II Ion Milling system) with an accelerating voltage of up 6 kV. The milling time was around 2 h for each sample. Coupled TGA was performed with a Netzsch STA‐449 F3 Jupiter instrument under a nitrogen flow at 20 mL min^−1^ as the carrier gas. A heating rate of 10 °C min^−1^ was applied between 25 and 700 °C. Two isothermal steps were included to simulate the synthesis conditions of the materials. The first isotherm at 30 °C for 20 min was selected to remove any remaining oxygen from the sample chamber, whereas the second isotherm at 450 °C for 20 min was applied to determine the mass loss during the crucial step of the synthesis. The porosity of the samples was analyzed using N_2_ physisorption isotherms measured at −196 °C on an Anton Paar QuantaTech Inc. iQ3 instrument (Boynton Beach, FL, USA). The samples were degassed under vacuum at 100 °C for 12 h before the measurement. The SSA of the samples was calculated using the Brunauer−Emmet−Teller equation applied to data points measured in the relative pressure range of 0.05 ≤ p/p_0_ ≤ 0.30. Pore size distribution (PSD) was calculated by applying the nonlocal density functional theory (NLDFT) kernel on the (metastable) adsorption branch, considering an amorphous silica surface and a spherical pore model.^[^ ^61^ ^]^ The total pore volume was determined from the relative pressure range of < 0.995. The calculations were carried out using ASiQwin 5.2 software provided by Anton Paar Quantatech Inc.

##### Electrochemical Testing

The as‐synthesized powders were ground using a mortar and pestle prior to the preparation of the slurry dispersion. For all electrode coatings, the following slurry formulation was used: active material (80 wt%), CMC (5 wt%), SBR (5 wt%), and carbon black C65 (10 wt%). DI was used as a solvent to prepare the slurries with a 25 wt% solid content. The components were mixed and homogenized at 3000 rpm for 5 min using a Thinky mixer. The obtained slurries were blade‐coated on a copper foil using a wet thickness of 60 μm. After coating, the electrodes were dried for 10 h at 60 °C in a vacuum oven. Hereafter, the electrodes were cut into 15 mm‐diameter disks and assembled into coin cells in half‐cell configuration (CR2032) in an Ar‐filled glove box (H_2_O and O_2_ < 1 ppm). Celgard 2325 with a 19 mm‐diameter disk was used as a separator, and 40 μl of 1M LiPF_6_ in EC/DMC with 10 wt% FEC electrolyte was used for each cell. Galvanostatic cycling was performed at 0.1 C‐rate, which was calculated using the theoretical capacity of SnS_2_ as 1230 mAh g^−1^. The cycling was performed in the potential windows between 0.01–1.3 V and 0.01–3.0 V versus Li/Li^+^, using a constant current constant voltage (CCCV) step protocol for the discharge. The constant current value during discharge was set for of 0.05 C as the cutoff during CCCV. CV was performed at a scan rate of 0.2 mV s^−1^ between 0.01 and 1.3 V versus Li/Li^+^. Galvanostatic intermittent titration (GITT) experiments were conducted during the first discharge cycle after each *x* = 0.35 or 50 mA g^−1^ GITT step pulsed at C/25 current density from Li_*x*_ (*x* ≈ 0) up to Li_*x*_ (*x* ≈ 8.4 or 1230 mAh g^−1^), with 2 h relaxation time between pulsing steps. The EIS measurements were programmed after each GITT step in the frequency range from 10 mHz to 100 kHz.

## Conflict of Interest

The authors declare no conflict of interest.

## Author Contributions

**Akzhan Bekzhanov**: conceptualization (lead); data curation (lead); formal analysis (lead); investigation (lead); methodology (lead); validation (lead); visualization (lead); writing—original draft (lead); writing—review & editing (lead). **Irshad Mohammad**: investigation (supporting); validation (supporting); writing—original draft (supporting). **Lukas Sallfeldner**: investigation (supporting); writing—original draft (supporting). **Freddy Kleitz**: data curation (supporting); resources (supporting); supervision (equal); validation (supporting); writing—review & editing (equal). **Damian Cupid**: funding acquisition (lead); project administration (lead); resources (lead); supervision (lead); validation (equal); writing—review & editing (equal).

## Supporting information

Supplementary Material

## Data Availability

The data that support the findings of this study are available from the corresponding author upon reasonable request.
