# Supplementary material for: One‐Step Solid‐State Synthesis of Sandwich‐like, Porous C–SnS2 Matrix Composites as Anode Materials for Rechargeable Lithium Ion Batteries
Source: Small Sci. 2025 Jul 7;5(9):2500192. doi: 10.1002/smsc.202500192 (PMC12412483; doi:10.1002/smsc.202500192)
Supplement: Supplementary file 1 — Supplementary Material [file SMSC-5-2500192-s001.pdf]

Supporting information

One-step solid-state synthesis of sandwich-like, porous C-SnS_2 matrix composites as anode materials for rechargeable lithium ion batteries.

Akzhan Bekzhanov[1, 2,3], Irshad Mohammed[1], Lukas Sallfeldner[2, 3], Freddy Kleitz[3], Damian Cupid[1]

[1] Center for Low-Emission Transport Vienna, Austrian Institute of Technology GmbH, 1210 Vienna, Austria;
[2]Vienna Doctoral School in Chemistry (DoSChem) University of Vienna Wahringer Str. 42, 1090 Vienna, Austria
[3]Department of Functional Materials and Catalysis, Faculty of Chemistry, University of Vienna, Währinger Str. 42, 1090 Vienna, Austria;

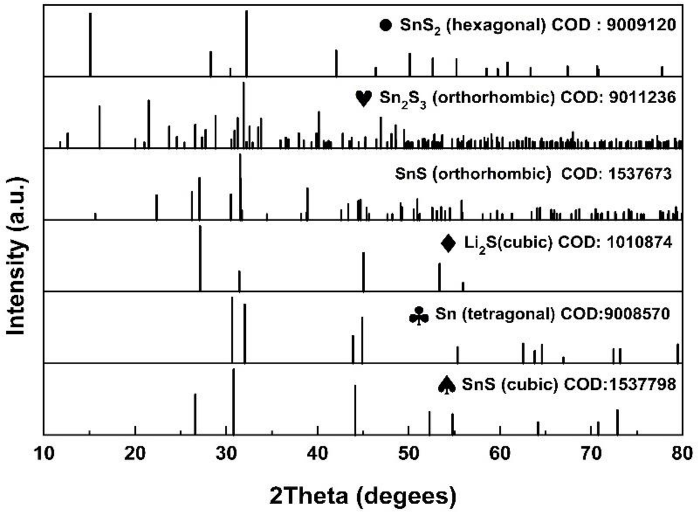

Figure S1. Reference XRD patterns.

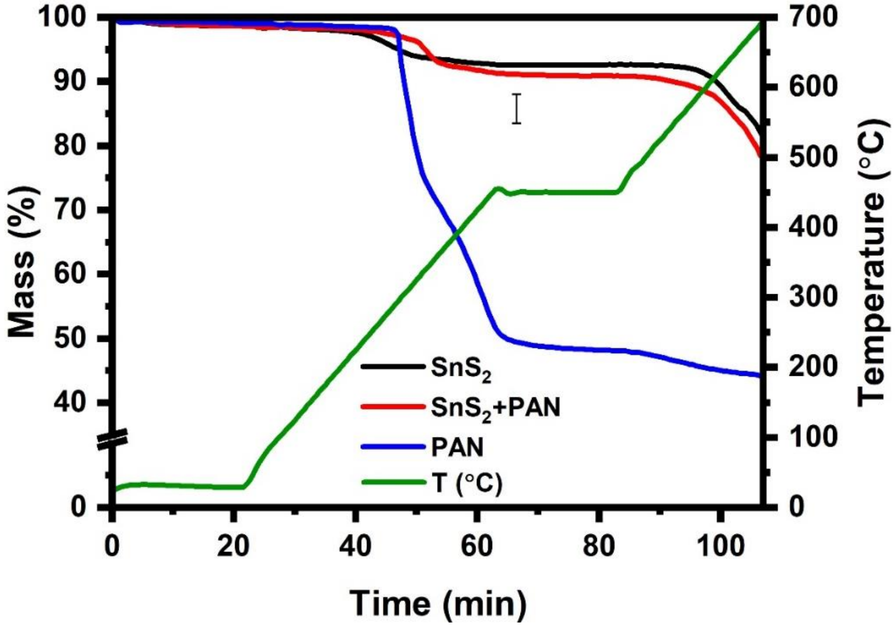

Figure S2. TGA results for SnS_2, SnS_2/PAN composite and PAN samples measured under N2 atmosphere.

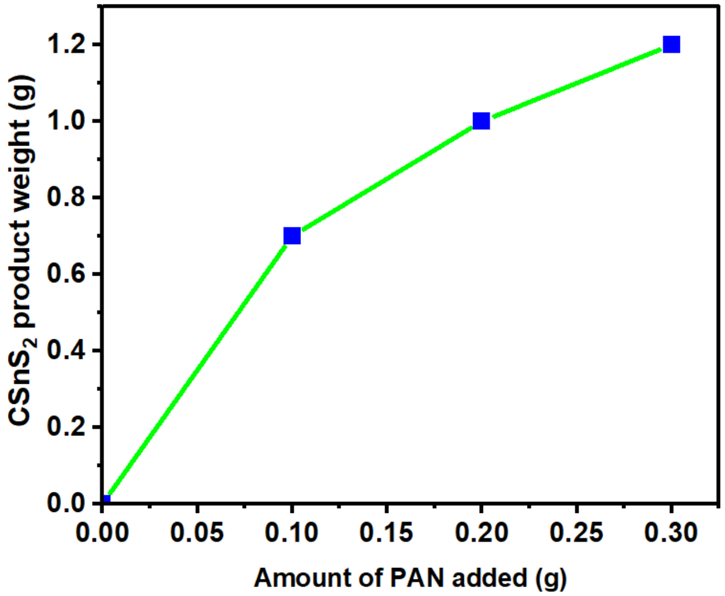

Figure S3. The graph illustrates the relationship between the weight of the C-SnS_2 product (g) obtained after synthesis and the amount of PAN precursor added (g).

Table S1. Mass loss of the samples based on TGA analysis.

**Sample**	**Mass loss (%) range 40-235°C**	**Mass loss (%) range 235-450°C**
SnS_2	1.3	4.8
SnS_2 (85wt.%) PAN (15wt.%)	1.4	7.7
PAN	0.6	51

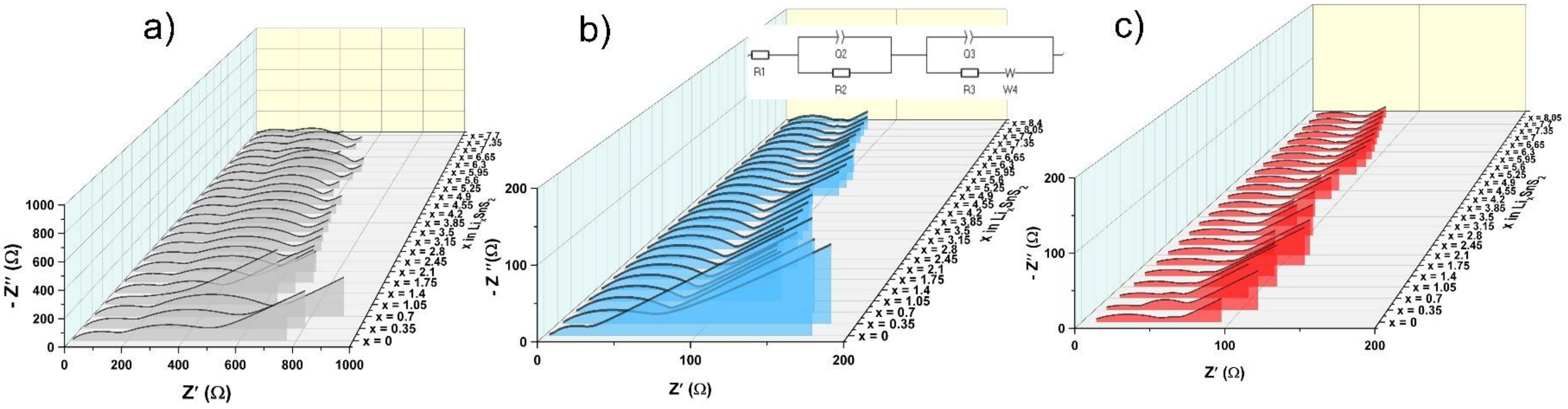

Figure S4. Fitted Nyquist plots of in situ EIS at first discharge cycle performed after each x=0.35 or 50mA g-1 GITT step pulsed at C/25 current density from Li_x (x=0) up to Li_x (x ≈ 8.4 or 1230 mAh g^{-1}) moles are shown for a) H-SnS_2, b) C-Sn_xS_y and c) C-SnS_2 samples.
